# Trehalose 6-Phosphate Regulates Photosynthesis and Assimilate Partitioning in Reproductive Tissue[Author-notes fn1] [Author-notes fn7]

**DOI:** 10.1104/pp.17.01673

**Published:** 2018-02-06

**Authors:** Maria Oszvald, Lucia F. Primavesi, Cara A. Griffiths, Jonathan Cohn, Shib Sankar Basu, Michael L. Nuccio, Matthew J. Paul

**Affiliations:** a Plant Science, Rothamsted Research, Harpenden, Hertfordshire, AL5 2JQ, United Kingdom; b Syngenta Crop Protection LLC, 9 Davis Drive, P.O. Box 12257, Research Triangle Park, North Carolina 27709

## Abstract

Transgenic maize (*Zea mays*) that expresses rice (*Oryza sativa*) *TREHALOSE PHOSPHATE PHOSPHATASE1* (*TPP1*) from the rice *MADS6* promoter, which is active over the flowering period, produces higher yields than wild type. This yield increase occurs with or without drought conditions during flowering. To understand the mechanistic basis of the increased yield, we characterized gene expression and metabolite profiles in leaves and developing female reproductive tissue, comprising florets, node, pith, and shank, over the flowering period with and without drought. The *MADS6* promoter was most active in the vasculature, particularly phloem companion cells in florets and pith, consistent with the largest decreases in trehalose 6-phosphate (T6P) levels (2- to 3-fold) being found in pith and florets. Low T6P led to decreased gene expression for primary metabolism and increased gene expression for secondary metabolism, particularly lipid-related pathways. Despite similar changes in gene expression, the pith and floret displayed opposing assimilate profiles: sugars, sugar phosphates, amino acids, and lipids increased in florets, but decreased in pith. Possibly explaining this assimilate distribution, seven *SWEET* genes were found to be up-regulated in the transgenic plants. SnRK1 activity and the expression of the gene for the SnRK1 beta subunit, expression of SnRK1 marker genes, and endogenous trehalose pathway genes were also altered. Furthermore, leaves of the transgenic maize maintained a higher photosynthetic rate for a longer period compared to wild type. In conclusion, we found that decreasing T6P in reproductive tissues down-regulates primary metabolism and up-regulates secondary metabolism, resulting in different metabolite profiles in component tissues. Our data implicate T6P/ SnRK1 as a major regulator of whole-plant resource allocation for crop yield improvement.

## Teaser

Decreased T6P in phloem cells of maize female reproductive tissue causes opposing changes in primary and secondary metabolism and a shift in assimilates within cobs in favor of florets, simultaneously increasing photosynthetic rate.

To avoid future food shortfalls, crop yields need to increase by more than is achievable at present by current crop improvement methods (Ray et al., 2013). It is also necessary to develop crops that are more stable in the face of increased climatic variability (Boyer et al., 2013). Hence, productivity combined with resilience is a sought-after goal. Improving crop performance under drought is complex, because the effects of water availability on crop yield depend on crop developmental stage and genetic factors. The flowering period is particularly sensitive to drought (Boyer and Westgate, 2004); restriction of water at this time can decrease seed set, final seed number, and harvested seed yield (Schussler and Westgate, 1991a, 1991b). Kernel abortion during drought at flowering can be alleviated by supplying Suc to reproductive tissue (Zinselmeier et al., 1995a, 1995b). Consequently, Suc metabolism in reproductive tissue has been proposed as a target to alleviate the effects of drought during the reproductive period (Boyer and McLaughlin, 2007). Rather than directly targeting Suc metabolism, it has been proposed that regulating the metabolism and utilization of Suc could be a more feasible target to alter assimilate partitioning (Boyer and McLaughlin, 2007).

The trehalose pathway is an important regulator of Suc utilization in plants (Schluepmann et al., 2003). Trehalose 6-phosphate (T6P), the precursor of trehalose, responds to Suc, likely as a signal of Suc availability (Lunn et al., 2006; Martinez-Barajas et al., 2011; Nunes et al., 2013a; Yadav et al., 2014). Altering levels of T6P causes changes in gene expression (Nunes et al., 2013a), plant metabolism (Zhang et al., 2009; Figueroa et al., 2016), and growth (Nunes et al., 2013a) such that metabolic reprogramming occurs in light of Suc availability. T6P can regulate starch levels through starch synthesis and breakdown (Kolbe et al., 2005; Martins et al., 2013) and enables the coordination of organic and amino acid metabolism with carbon availability (Figueroa et al., 2016). Such whole-scale effects are likely to be mediated by signal transduction and interaction with the feast/famine protein kinase, SnRK1 (Zhang et al., 2009; Delatte et al., 2011; Nunes et al., 2013a, 2013b; Tsai and Gazzarrini, 2014). By mediating such effects on metabolism, growth, and development, T6P ensures effective use of Suc in addition to maintaining Suc homeostasis. Yadav et al. (2014) have put forward a theory of the T6P:Suc nexus; T6P levels could alter both the use and allocation of Suc by increasing gene expression for the use of Suc and mediating allocation by perturbing Suc homeostasis. There have been many reports of associations between the trehalose pathway and drought tolerance but no detailed mechanistic basis for such a correlation. The abundance of trehalose itself is too low to provide osmotic or oxidative stress protection against desiccation. Constitutive expression of trehalose pathway transgenes to alter T6P accumulation has produced examples of improved drought tolerance, but this may be because of reduced growth, which decreases water loss and improves survival, and does not improve productivity as is required in agriculture (Romero et al., 1997; Cortina and Cuiliáñez-Macià, 2005).

Nuccio et al. (2015) targeted changes in T6P abundance in reproductive tissue during the flowering period using a rice (*Oryza sativa*) *MADS6*: trehalose phosphate phosphatase (*OSMADS6: TPP1*) construct to alter Suc metabolism for yield preservation during drought. This modification simultaneously decreased T6P and increased Suc in female florets 5 d before pollination (Nuccio et al., 2015). In extensive field trials over several years and locations, the transgenic maize (*Zea mays*) demonstrated significantly improved yield, with and without drought during the flowering period, through enhanced kernel set. This provides one of very few reports wherein transgenic technology that has modified an intrinsic plant process has substantially improved yield and was reproducible in the field environment. Despite the importance of this success, little is known of the mechanistic details that underpin this yield improvement.

In this study, we performed a detailed analysis of plants that were higher yielding than wild type in the field trials of Nuccio et al. (2015). We started from existing models of the mode of action of T6P through SnRK1. Reproductive tissue was sectioned into ear florets (female reproductive structures), pith (the vascular core of the ear), node (the vasculature in the stalk where the ear emerges), and shank (the branch where the ear attaches to the stalk). *OSMADS6: TPP1* expression was found to be associated with phloem tissue in these structures, and transgene expression was greatest in florets and pith. Consistent with decreased T6P concentrations, primary metabolic pathways were down-regulated, and secondary metabolic pathways were up-regulated in the tissues where *OSMADS6* promoter drove GUS expression. This altered the distribution in the component tissues of the reproductive structures away from pith toward the ear florets. *SWEET* genes were the only class of gene associated with assimilate transfer that were consistently affected in pith and florets. There were also changes in the expression of SnRK1 marker genes, endogenous trehalose pathway genes, and the gene encoding the SnRK1 beta subunit. Leaves of transgenic plants maintained higher rates of photosynthesis for longer during the reproductive period. Our results provide evidence that T6P/ SnRK1 acts as a central regulator of the balance between primary and secondary metabolism, assimilate distribution and the whole-plant source-sink interaction for crop yield improvement.

## RESULTS

### Expression of *OSMADS6: TPP1* During the Flowering Period

**Figure 1.**
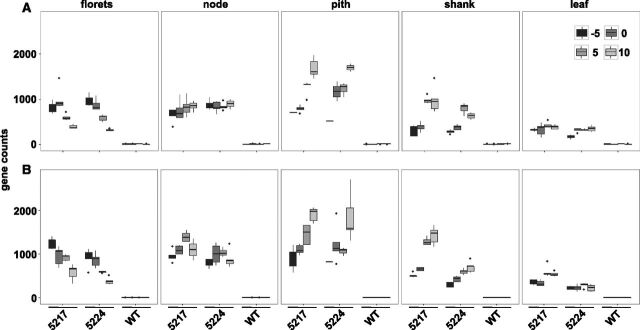
*OSMADS6: TPP1* expression in reproductive tissues and leaves during early reproductive development. Data are from US (A) and DS (B) plants. Normalized gene count data are plotted for each transgenic event (5,217 or 5,224) and the wild-type (WT) A188 line. The horizontal line in each box indicates the mean. Vertical lines indicate the range for each sample. The time points are 5 d before pollination (−5), day of pollination (0), 5 d after pollination (5), and 10 d after pollination (10). Data are the mean ±SD of 5 biological replicates.

**Figure 2.**
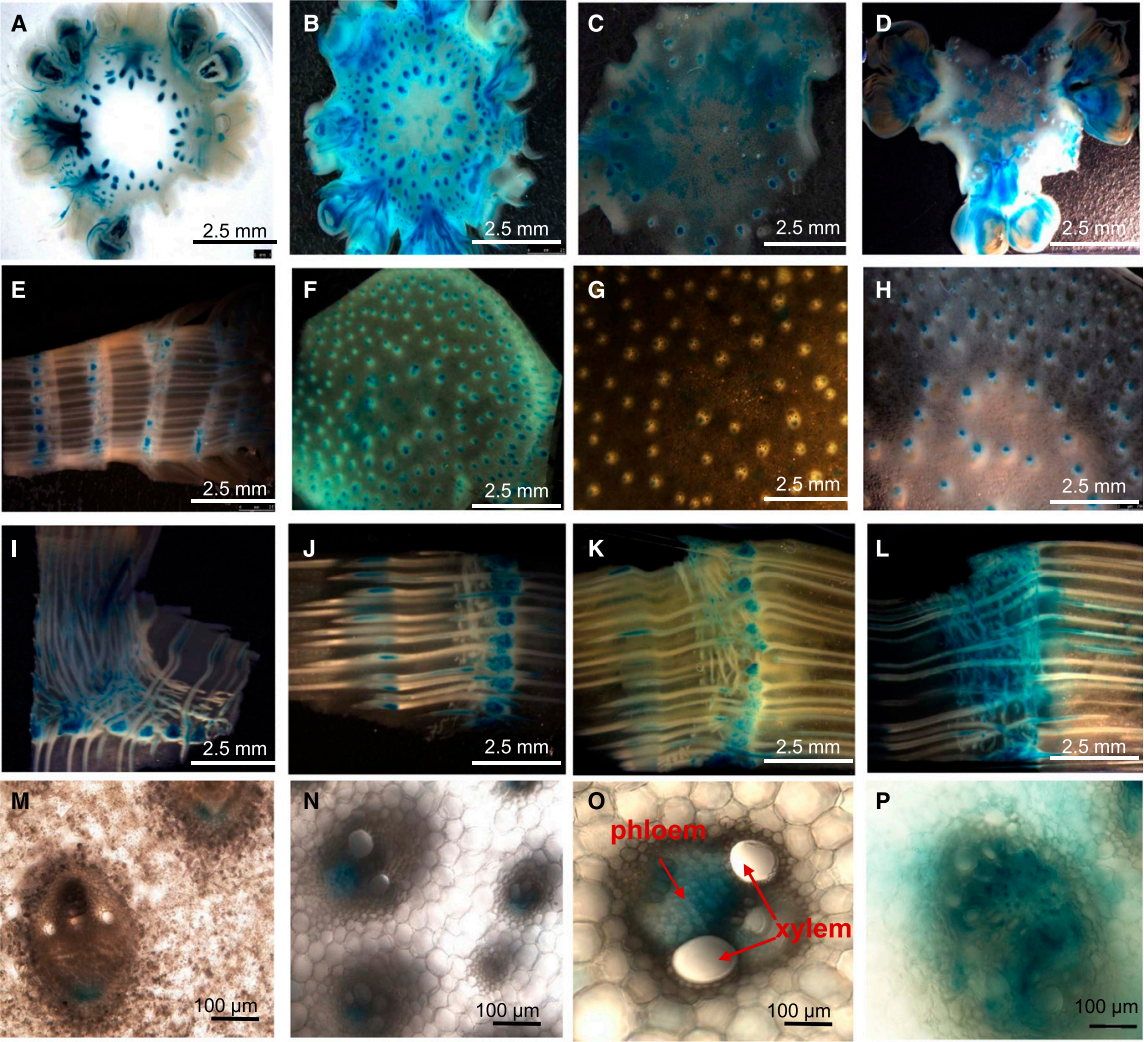
Histochemical localization of GUS activity produced by the *OSMADS6: GUS* reporter gene in US maize during early reproductive development. Samples were collected from ear florets (A–D), shank (E–H), node (I–L), shank at high magnification (M–N), node at high magnification (O), and pith at high magnification (P). Samples were collected at 5 d before pollination (A, E, I, M), day of pollination (B, F, J, N), 5 d after pollination (C, G, K, O), and 10 d after pollination (D, H, L, P). Samples were incubated in the histochemical reagent and cleared as described in the “Methods.” Xylem and phloem cells are indicated by the red arrows.

Female reproductive and leaf tissues were sampled for profiling analysis at 5-d intervals beginning at silk emergence, 5 d before pollination (−5), under controlled environmental conditions. Expression of *OSMADS6: TPP1* reached its highest levels in pith tissue compared to node, shank, and floret (Fig. 1, A and B). There were different *OSMADS6: TPP1* trends with respect to development in each tissue with expression increasing over time in shank and pith but decreasing over time in florets. Activity was most constant in the node during development. Analysis of *OSMADS6: GUS* shows *OSMADS6* expression localized to vasculature in all tissues, and phloem companion cells in particular (Fig. 2, Supplemental Figs. S1 and S2). Figure 1 shows that *OSMADS6: TPP1* transcript was detected in leaves, but when leaf tissue from *OSMADS6: GUS* plants was evaluated for GUS activity by histochemical analysis, no evidence of enzyme activity was found (Supplemental Fig. S1). Hence, *OSMADS6* does not appear to direct protein expression in leaves.

### T6P and Trehalose

**Figure 3.**
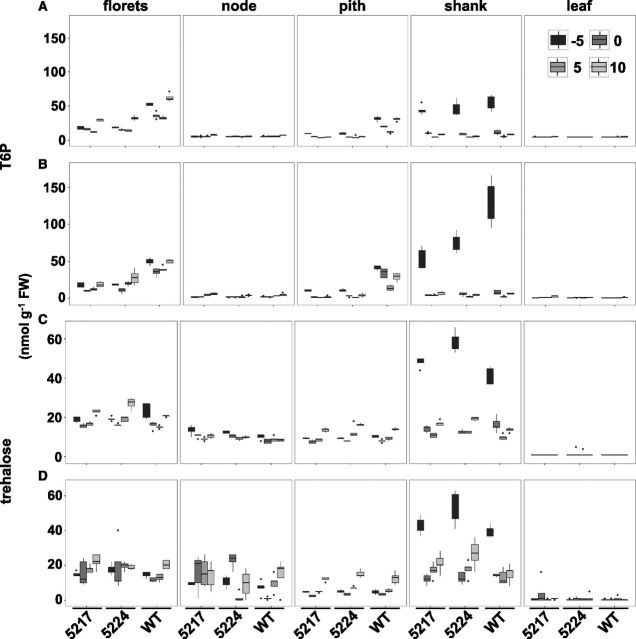
Effect of the *OSMADS6: TPP1* on T6P and trehalose in reproductive tissues and leaves during early reproductive development. Samples were collected from US (A and C) and DS (B and D) plants at 5 d before pollination (−5), day of pollination (0), 5 d after pollination (5), and 10 d after pollination (10). Vertical lines indicate the range for each sample. Data are the mean ± sd (*n* = 5).

In wild-type reproductive tissue under well-watered (unstressed [US]) conditions, T6P abundance ranged from 4 nmol g^−1^ fresh weight (FW) in the node, where it was most stable during development compared to other reproductive tissues, to 60 nmol g^−1^ FW in the shank 5 d before pollination (−5; Fig. 3A). T6P then fell to <10 nmol g^−1^ FW over the next 15 d (0, 5, 10). Overall during development, T6P levels were highest in florets and pith, between 32 and 60 nmol g^−1^ FW and 11 and 30 nmol g^−1^ FW, respectively. T6P levels in leaves were around 5 nmol g^−1^ FW. In shank, the large peak of T6P 5 d before pollination was increased by drought to 102 nmol g^−1^ FW. Drought also increased T6P in pith, but decreased it at later time points in florets. *OSMADS6: TPP1* resulted in 2- to 3-fold less T6P in pith and floret tissue under both well-watered and drought conditions. The transgene barely affected T6P levels in node and leaves and increased T6P only 2-fold at the first time point in shank tissue. Trehalose content followed the same pattern as T6P for tissue type and developmental stage, with a 40 nmol g^−1^ FW peak of trehalose in shank tissue at day −5 (Fig. 3C). Drought increased trehalose in node (Fig. 3D). *OSMADS6: TPP1* had little effect on trehalose, increasing it slightly in node but decreasing it in pith and florets. Trehalose was barely detectable in leaves.

### Metabolite Profiling

**Figure 4.**
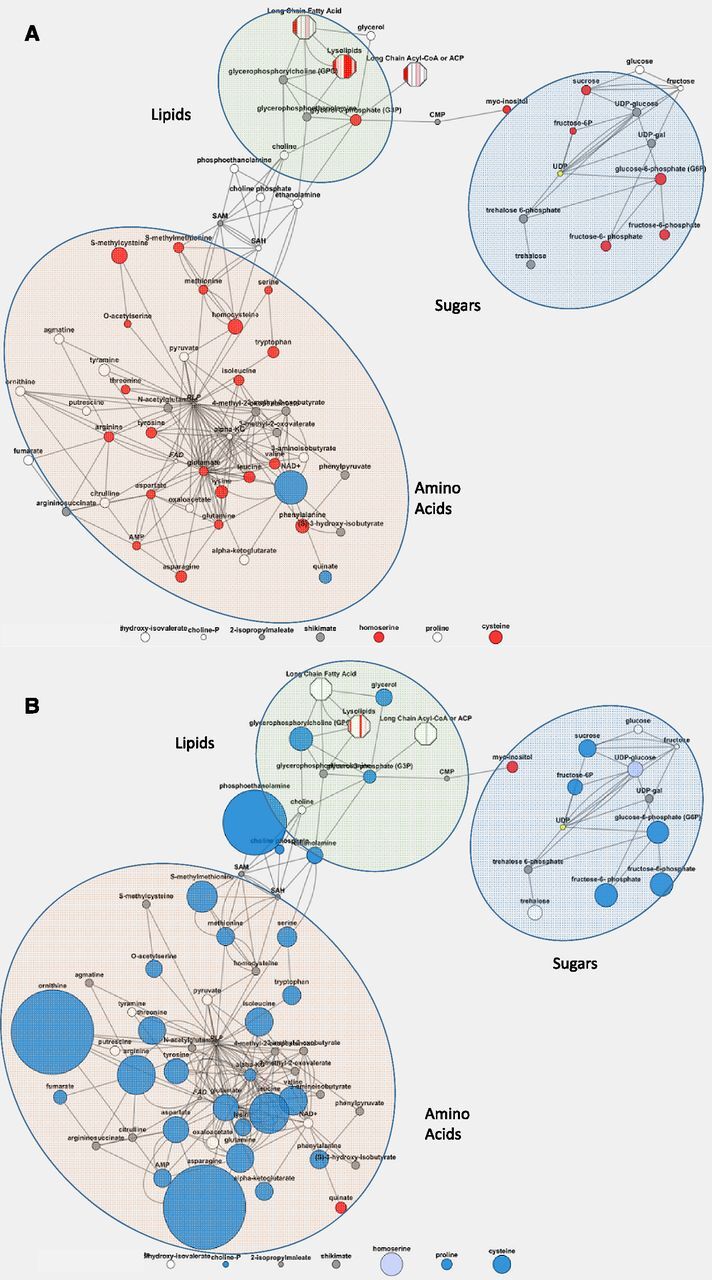
Screen shot from Metabosync showing change in metabolite abundance in response to *OSMADS6: TPP1*. Representative contrasts of transgenic event 5,224 compared to wild type grown in US conditions to show full extent of metabolite changes. A, Floret tissue at day of pollination. B, Pith tissue 5 d after pollination. Biochemicals shown are key metabolites in aromatic amino acid metabolism, Glu family amino acid synthesis, phospholipid metabolism, Suc metabolism, Asp family amino acid synthesis, Arg biosynthesis, BCAA metabolism, trehalose biosynthesis, and nitrogen metabolism. Circles represent metabolites as labeled. The size of the circle indicates statistical significance of difference of mean values from Welch’s T-tests. Blue indicates fold change (ratio of mean value from all replicates in samples of transgenic event/ wild type) of <1 and red indicates fold change of >1.

The largest effects of *OSMADS6: TPP1* on metabolite profiles were found in floret and pith under well-watered and drought conditions (Fig. 4, A and B; Supplemental Fig. S3, A and B), which coincided with the largest expression of transgene and largest effect of the transgene on T6P (Fig. 3, A and B). The same trends were observed under well-watered and drought conditions. The changes in metabolites in floret and pith were largely in opposite directions, particularly for Suc, Glc, and Fru, and amino acids, which were increased in florets but decreased in pith, although amino acids in floret were unchanged or decreased at the last two time points. Levels of Xyl and xylulose were decreased in both tissues. AMP increased in florets and decreased in pith in well-watered conditions. Contents of phospholipids, sphingolipids, and sterols were increased in florets throughout development and were unchanged or decreased in pith. Node tissue was similar to pith with regard to amino acids, but dissimilar to pith for sugars which increased in node. Patterns of changes were less clear for shank and leaf, although leaves of transgenic plants had more carbohydrates than wild type. Allantoin was the only metabolite that increased in all tissues in transgenic plants. The known link between T6P and Suc (Nunes et al., 2013a; Yadav et al., 2014) was broken in floret and node where a decrease in T6P (Fig. 3, A and B) was related to more Suc. Increased Suc in leaves did not correspond to more T6P. In pith and shank, however, less T6P (Fig. 3, A and B) was related to less Suc (Supplemental Fig. S3, A and B).

### Gene Expression

**Figure 5.**
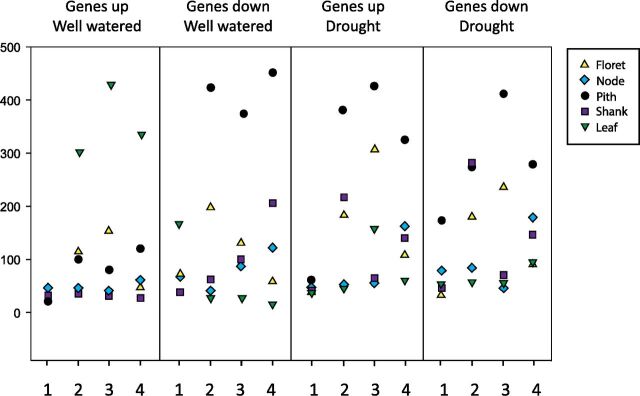
Genes significantly up-regulated or down-regulated in different tissues in both *OSMADS6: TPP1* transgenic lines compared to wild type in well-watered conditions and under drought. The time points are 5 d before pollination (1), day of pollination (2), 5 d after pollination (3), and 10 d after pollination (4).

**Figure 6.**
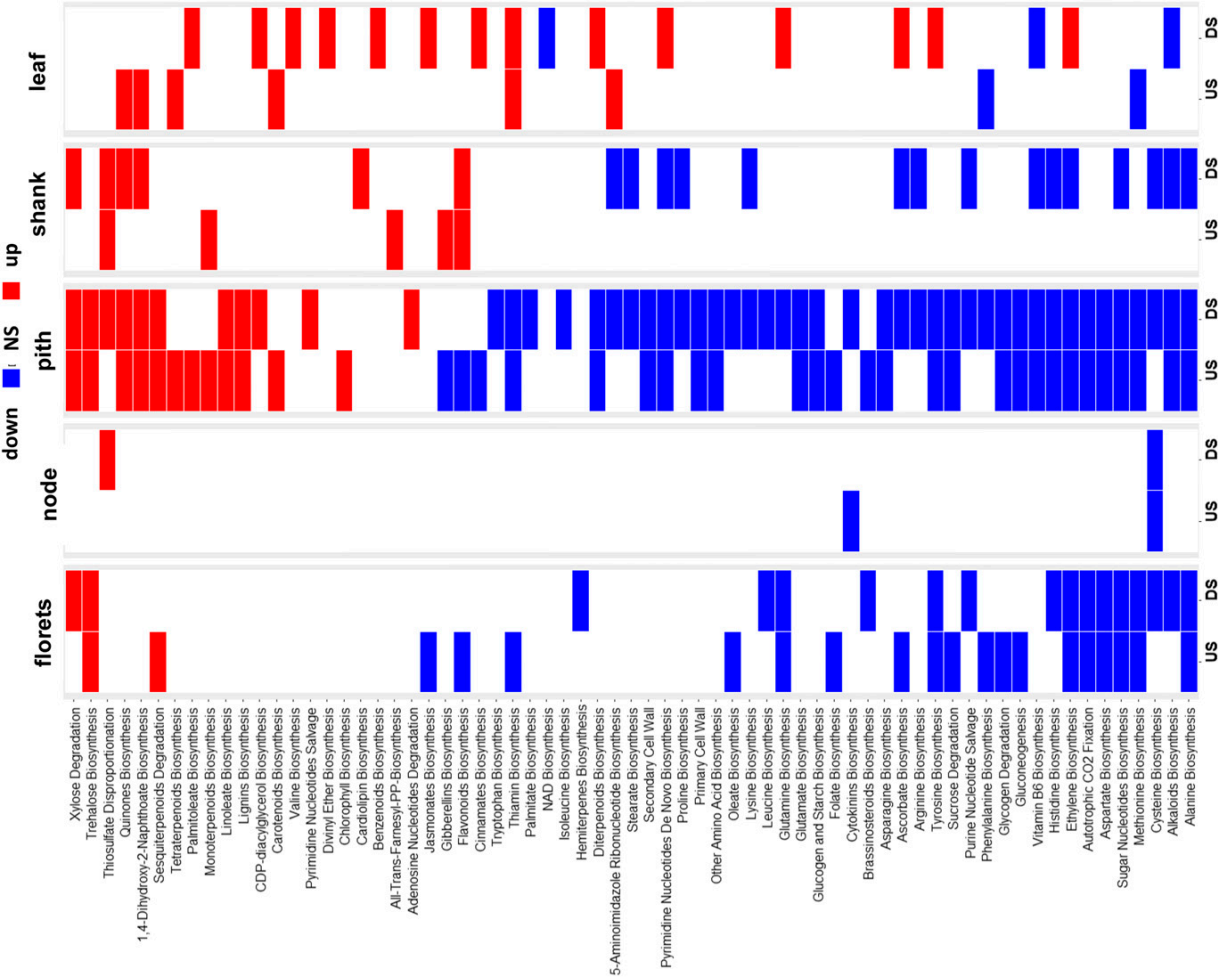
*OSMADS6: TPP1* effect on biochemical pathways. Transcript profiling data from both the 5,217 and 5,224 events were compared to wild type (A188) to identify significantly perturbed biochemical pathways. A white cell indicates pathways that were not affected, and a dark cell (red, up; blue, down) indicates pathways that were significantly affected by the transgene. The data are from US and DS plants. Results from the four time points were condensed by only reporting pathways significantly affected in at least two time points.

Gene expression analysis from RNAseq analyses can be summarized by categorizing genes significantly increased or decreased in both transgenic lines compared to wild type in all tissues under well-watered and drought conditions (Fig. 5; Supplemental Tables S1–S4). The Roast algorithm was used to identify biochemical pathways significantly perturbed (Fig. 6). Overall, transgenic pith tissue exhibited the greatest changes in gene expression due to transgene followed by floret, shank, node, and leaf. There were no large differences between well-watered and drought conditions in numbers or types of genes affected. A total of 67 pathway categories were perturbed by the transgene in both events at two or more time points in at least one tissue in either US or DS plants (Fig. 6). Overall, expression of primary metabolic pathways (major flux e.g. sugar nucleotides, starch, cell wall, amino acids) was decreased, particularly in pith and floret, whereas expression of genes for secondary metabolic pathways (minor flux e.g. lipids, trehalose) was increased (Fig. 6).

### SnRK1 Marker Gene Expression

**Figure 7.**
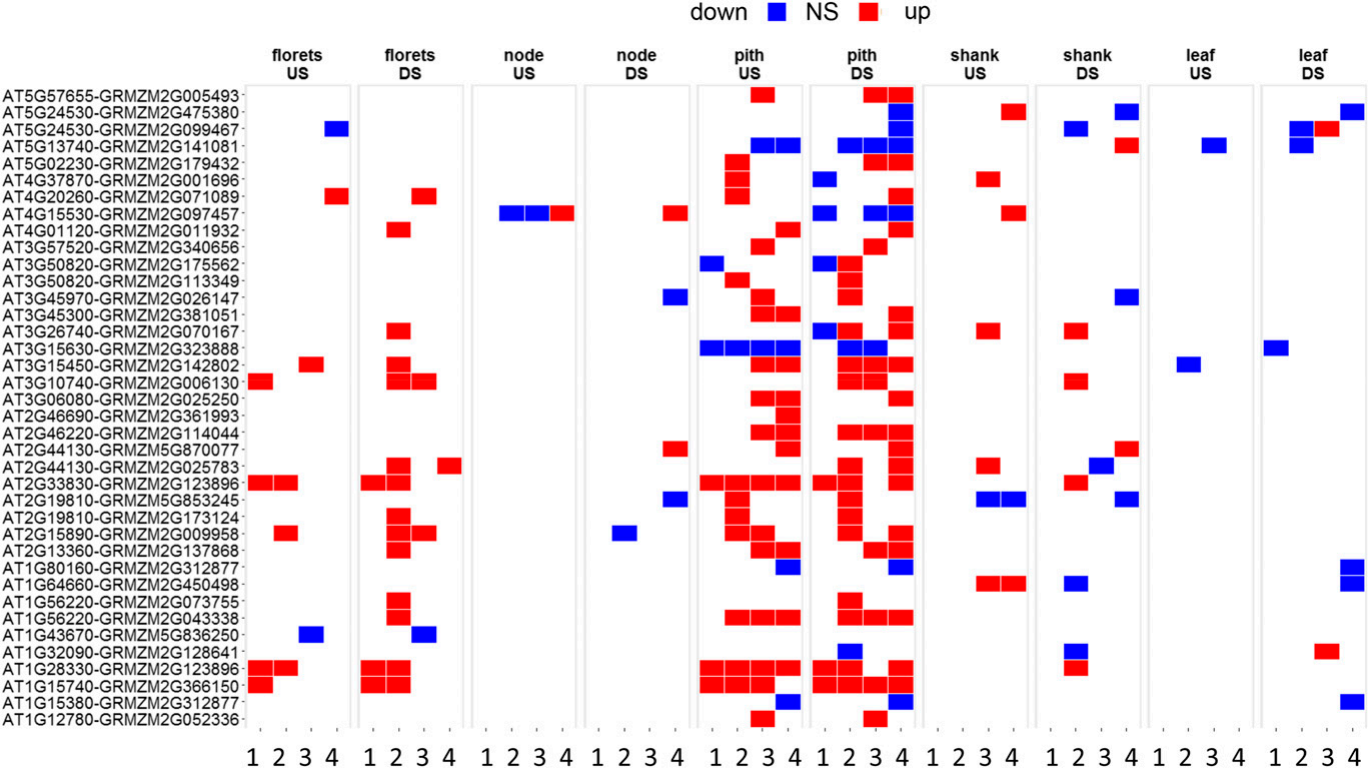

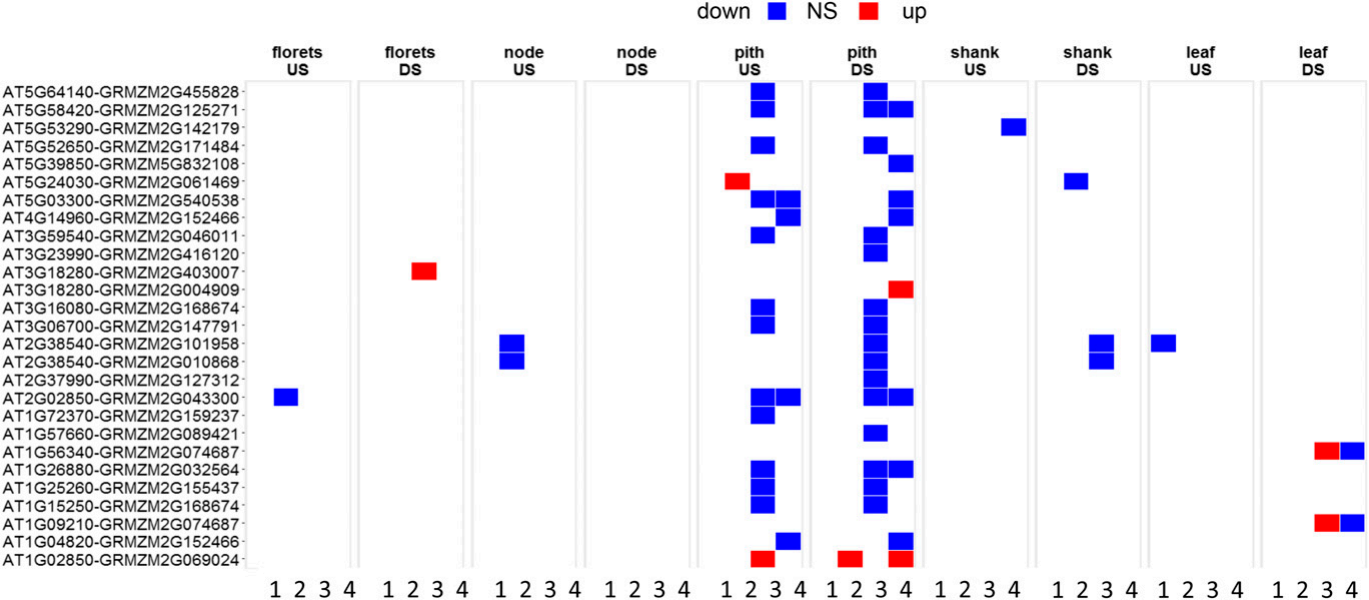
Heat map showing the effect of *OSMADS6: TPP1* on SnRK1-regulated genes. Maize orthologs of Arabidopsis/wheat genes shown to be induced (A) or suppressed (B) by SnRK1 in Arabidopsis/wheat (Zhang et al., 2009; Martinez-Barajas et al., 2011) were examined using differential expression analysis from US or DS plants between wild-type and transgenic lines 5,217 and 5,224. Red indicates up-regulation and blue indicates down-regulation relative to wild type (A188). White indicates no significant (NS) difference and gray indicates no data. The time points are 5 d before pollination (1), day of pollination (2), 5 d after pollination (3), and 10 d after pollination (4).

Given the known effects of SnRK1 on metabolic pathways (Zhang et al., 2009; Figueroa et al., 2016), we quantified the extent of changes in SnRK1 marker gene expression previously identified in Baena-González et al. (2007), Zhang et al. (2009), and Martinez-Barajas et al. (2011). Maize orthologs of Arabidopsis (*Arabidopsis thaliana*) genes reported to be regulated by SnRK1 were established using a combination of OrthoMCL, reciprocal best BLAST match and gene synteny (Li et al., 2003). Putative maize orthologs (183 induced markers, 242 repressed markers) are reported in Supplemental Tables S5, A and B. Of those that changed in both lines on at least one time point, 23 orthologs of SnRK1 markers normally induced by SnRK1 were induced under US conditions in pith and 25 were induced under drought conditions (DS; Fig. 7A). Also in pith, 14 maize orthologs of Arabidopsis genes reported to be repressed by SnRK1 were repressed in both events in at least one time point tested under US and 19 under DS conditions (Fig. 7B). Changes in SnRK1 markers were also observed in florets (Fig. 7A; Supplemental Table S5B).

### SnRK1 Activity and Gene Expression

**Figure 8.**
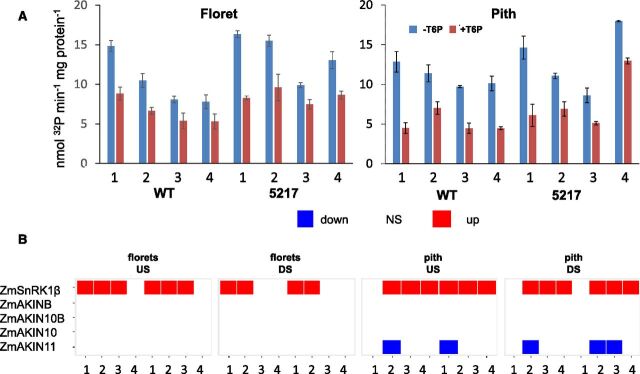
Effect of *OSMADS6: TPP1* on SnRK1 activity and gene expression. Pith and floret tissues were examined for (A) extractable SnRK1 activity (line 5127) and (B) differential expression of maize orthologs of Arabidopsis genes that encode SnRK1 subunits between wild type and transgenic line 5,217 and 5,224. SnRK1 activity was assayed in the presence and absence of 1 mm T6P. Data are the mean ± sd (*n* = 3). The time points are 5 d before pollination (1), day of pollination (2), 5 d after pollination (3), and 10 d after pollination (4).

In US plants, SnRK1 activities were higher in transgenic floret tissues compared to wild type at day 0 and day 10 (Fig. 8A; Supplemental Table S6). In pith, SnRK1 activity was higher in transgenics compared to wild type at day 10. *SnRK1β* expression increased in transgenics compared to wild type in pith and floret tissue, whereas expression of the gene encoding the SnRK1 AKIN11 subunit decreased in pith (Fig. 8B). SnRK1 activity was inhibited by T6P in floret and pith tissues (Fig. 8A). Combined with decreases in T6P (Fig. 3), it is likely that in vivo activities of SnRK1 were significantly higher in transgenics compared to wild type.

### Trehalose Pathway

**Figure 9.**
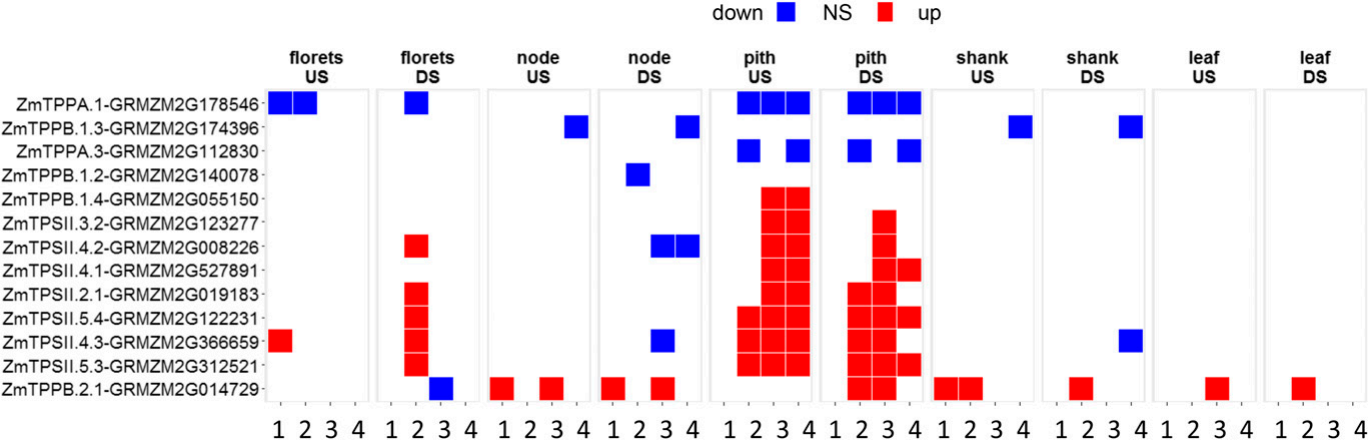
Effect of *OSMADS6: TPP1* on trehalose metabolism gene expression. Differential expression analysis of TPS and TPP gene family members between wild-type and transgenic lines 5,217 and 5,224. The time points are 5 d before pollination (1), day of pollination (2), 5 d after pollination (3), and 10 d after pollination (4).

The endogenous trehalose biosynthetic pathway was one of the few biosynthetic pathways to be up-regulated in transgenics compared to wild type (Fig. 6). The gene set defined by Henry et al. (2014) was analyzed in more detail. There were large changes in pith and, to a lesser extent, florets (Fig. 9; Supplemental Table S7) in well-watered (US) and DS tissues. Genes encoding class II trehalose phosphate synthase (TPS) and TPPB were induced in transgenics compared to wild type in well-watered and drought conditions, whereas *TPPA* was repressed. *TPS1* was also induced in pith.

### *SWEET* Genes

**Figure 10.**
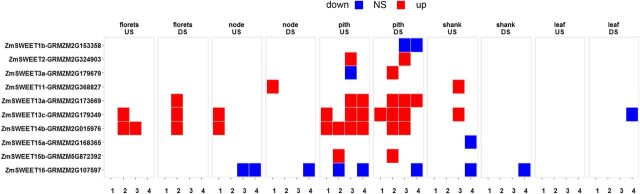
Heat map showing the effect of *OSMADS6: TPP1* on SWEET genes. Heat maps represent differential expression analysis between wild-type and transgenic lines 5,217 and 5,224. The time points are 5 d before pollination (1), day of pollination (2), 5 d after pollination (3), and 10 d after pollination (4).

**Figure 11.**
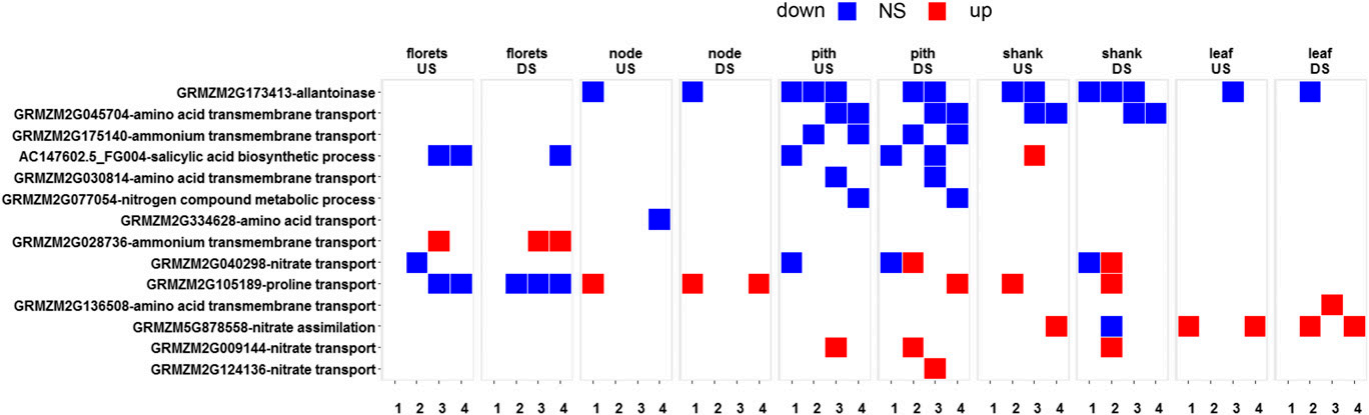
Heat map showing allantoinase and nitrogen transport and metabolic processes. Heat maps represent differential expression analysis between wild-type and transgenic lines 5,217 and 5,224. The time points are 5 d before pollination (1), day of pollination (2), 5 d after pollination (3), and 10 d after pollination (4).

We next sought to explain changes in the distribution of assimilate between pith and floret. We found that *SWEET* genes were the only class of genes involved in transport or efflux that had significantly altered expression levels (Fig. 10). Seven *SWEET* genes were up-regulated across the reproductive tissue, with greatest changes in pith (Fig. 10; Supplemental Table S8). No significant changes in expression were found for genes encoding Suc transporters; amino acid transporter gene expression decreased, whereas expression of genes for two nitrate transporters increased in pith (Fig. 11).

### Allantoinase

Given the consistent change in allantoin levels in most of the transgenic samples, expression of genes involved in its metabolism was examined. Allantoinase, which metabolizes allantoin to allantoate, was decreased in all tissue without drought except florets, and in all tissues under drought conditions (Fig. 11). Changes were compared to other genes putatively involved in nitrogen metabolism, none of which changed as consistently as allantoinase.

### Photosynthesis

**Figure 12.**
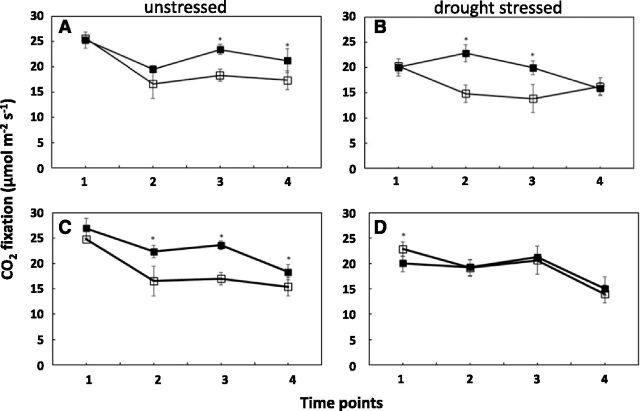
Rates of photosynthesis during 2-week flowering period. Wild type (white symbols) and *OSMADS6: TPP1* transgenic line 5,224 (black symbols). A and B, The leaf next to the ear and (C and D) the leaf above the ear. US (A and C) and DS (B and D). Time points are 5 d before pollination (1), day of pollination (2), 5 d after pollination (3), and 10 d after pollination (4). Rates of CO_2_ uptake were measured under the growing conditions (ambient CO_2_ (400 µL L^–1^), leaf temperature 27°C, photosynthetic photon flux density 600 µmol m^–2^ s^–1^, relative air humidity 60 ± 5%. Data are the mean ± sd (*n* = 4). *Statistical significance between transgenic and wild type at *P* < 0.05.

**Figure 13.**
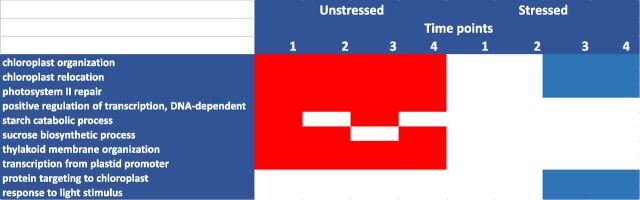
Heat map showing photosynthesis genes in mature leaves. Heat maps represent differential expression analysis between wild type and transgenic lines 5,217 and 5,224. The time points are 5 d before pollination (1), day of pollination (2), 5 d after pollination (3), and 10 d after pollination (4).

Rates of CO_2_ uptake were determined in the leaf adjacent to the developing cob and the leaf above the developing ear under both well-watered and drought conditions. CO_2_ uptake was up to 54% higher in transgenics compared to wild type (Fig. 12), with the largest percentage increase in primary leaf under drought (Fig. 12B). Effects were greatest at day 0 and day 5. There were no differences between transgenic and wild type in secondary leaves under drought (Fig. 12D). Analysis of gene expression in primary leaves showed up-regulation of genes related to chloroplast and Suc biosynthesis in US tissue and decreases in chloroplast processes in drought-stressed leaves (Fig. 13).

## DISCUSSION

Expression of a trehalose pathway gene linked to the rice *MADS6* promoter (*OSMADS6: TPP1*) in maize reproductive tissue was previously shown to increase maize yield in the field both with and without drought during the 2- to 3-week flowering period (Nuccio et al., 2015). This is one of the very few reports wherein genetically modifying an intrinsic process increases yield in field conditions. It is important to determine the biochemical and molecular basis of the yield increase to inform and develop crop improvement strategies. Four main conclusions can be drawn from this study. Firstly, localization of *OSMADS6: TPP1* transgene expression in phloem cells of pith and florets lowered T6P levels 2- to 3-fold. This resulted in the down-regulation of primary metabolic pathways and up-regulation of secondary metabolism, particularly lipid-related pathways, most strongly in pith. Secondly, this *OSMADS6: TPP1* transgene expression resulted in opposing changes in metabolites: increased sugars, amino acids, and lipids in florets but decreased levels in pith; altered *SWEET* gene expression could account for assimilate transfer from pith toward floret. Allantoin was the only metabolite to increase in all tissues associated with the down-regulation of allantoinase transcript in transgenic lines. Thirdly, a higher leaf photosynthetic rate in transgenics was likely to be in response to the enhanced movement of assimilate to florets. Fourthly, changes in SnRK1 activity and in the expression of its subunit genes as well of SnRK1 marker genes and endogenous trehalose pathway genes implicate T6P/ SnRK1 as a central mechanism of assimilate partitioning, allocation, and source-sink regulation in crops.

### Decreasing T6P in Reproductive Tissue Down-Regulates Primary Metabolism and Up-Regulates Secondary Metabolism, but Causes Distinct Changes in Metabolite Profiles in Component Tissues

Following the current thinking on the role of T6P in plants, a decrease in T6P would be expected to repress the biosynthetic activity of metabolic pathways (Zhang et al., 2009; Figueroa et al., 2016). This is observed in the reproductive tissues in this study with regard to primary metabolism (sugar nucleotide, amino acids, starch, and cell wall; Fig. 6). However, an increase in the activity of the pathways of lipid biosynthesis was observed. This represents a refinement of the earlier hypothesis in that low T6P does not put a brake on all metabolic pathways but may stimulate secondary metabolism, lipids in this case, and hence produce qualitative changes in metabolism. Despite similarities in gene expression between component parts of reproductive tissue, metabolite profiles showed contrasting patterns in pith and florets, with pith having reduced assimilate content and floret increased assimilate content (Fig. 5, A and B; Supplemental Fig. S3, A and B). This may be explained because pith is composed of soft spongy parenchyma cells that store and transport nutrients to the developing florets, which are terminal sinks for assimilate. Hence, while reproductive tissue is an overall sink for assimilate, within the developing cob, pith is a source of assimilate for florets. Effects of T6P are likely to be highly context specific. This is evident in the different response of Arabidopsis seedlings compared to rosettes, which are mainly sink tissue and source tissue, respectively (Zhang et al., 2009; Wingler et al., 2012). Previous studies have shown an association of trehalose pathway gene expression with vasculature (Ramon et al., 2009), and the expression of *Attps1* is high in phloem companion cells (Genevestigator, https://genevestigator.com/gv/). Vascular expression of *TPS1* resulted in early flowering, suggesting a biological significance of the pathway in vascular tissue (Ruiz-Salas et al., 2016). This, combined with the T6P-associated changes to assimilate distribution in phloem cells found in this study, suggest that T6P may regulate the flow of assimilates between tissues such as the pith and floret of maize reproductive structures. Regulation of *SWEET* genes could be a key factor in the regulation of T6P-mediated assimilate transfer, as it was the only major class of protein associated with assimilate transfer to be consistently affected in this study (Fig. 10). Five *SWEET* genes were up-regulated in pith, potentially implicating them in the movement of Suc from pith to floret (Fig. 10); *SWEET* genes are proposed to efflux Suc to the phloem apoplasm (Braun et al., 2014). In particular, *SWEET13*, which was strongly affected in our study, has been previously singled out as a target for crop improvement in maize (Bezrutczyk et al., 2017). In explaining elevated amino acids in florets in the absence of consistent up-regulation of amino acid synthesis or amino acid transport genes, it is possible that increased Suc availability may have resulted in more carbon skeleton for the synthesis of amino acids. Allantoin was the only metabolite to be increased consistently in all tissues (Supplemental Fig. S3, A and B) found to be associated with a decrease in transcript for allantoinase (Fig. 11). Allantoin is associated with nitrogen assimilation in legumes and is considered a transport form of nitrogen (Coneva et al., 2014; Collier and Tegeder, 2012). It is possible that this may be associated with changes in amino acids, although the mechanistic basis of this is not known. Allantoin accumulation is associated with a number of traits involved in the tolerance of stresses, such as cadmium and salt, through antioxidant mechanisms (Brychkova et al., 2008), and jasmonic acid and ABA signaling (Takagi et al., 2016).

### Photosynthesis Rate Is Maintained for Longer in Leaves of Transgenics

The normal developmental decline of photosynthesis in leaves was slowed in the transgenics (Fig. 12). Since there was no evidence of expression of the transgene in leaves, explanations for elevated photosynthesis must come from elsewhere. It is possible that greater movement of Suc from pith into florets increased demand for Suc, which maintained a higher photosynthetic rate for longer. Up-regulation of a number of *SWEET* genes in reproductive tissue, including node and shank (Fig. 10), may have mediated the enhanced flow of Suc from leaves. Sink regulation of photosynthesis has been a long-observed phenomenon (Paul and Foyer 2001). Recent work strongly implicates SWEET proteins in source-sink relationships in Arabidopsis (Durand et al., 2017). Suc levels in leaves of transgenics were also increased. This was consistent with increases in gene expression for Suc biosynthesis and chloroplast processes in leaves (Fig. 13) and provides evidence of a wider role for T6P in source-sink relations regulating metabolism in sinks, which can provide feedforward regulation of leaf photosynthesis

### T6P/SnRK1-Mediated Regulation

A second part of the current model of T6P’s mode of action is that SnRK1 mediates changes in gene expression associated with T6P abundance (Zhang et al., 2009; Nunes et al., 2013a). To better understand whether SnRK1 could mediate the effects of lower T6P, expression of SnRK1 marker homologs was determined. Despite the fact that over the long term, direct cause-and-effect correlations are likely to be weakened by secondary and tertiary effects, there was evidence that changes in T6P resulted in altered regulation of gene expression through SnRK1, as the SnRK1 marker genes normally induced by SnRK1 were induced (Fig. 7A) and those repressed by SnRK1 were more repressed than wild type (Fig. 7B). SnRK1 was also found to be inhibited by T6P, and its activity, in the absence of T6P, was elevated in transgenic pith and florets (Fig. 8). These data provide in vivo and in vitro evidence of increased SnRK1 activity as a consequence of decreased T6P. This was associated with altered expression of the gene for the SnRK1 beta subunit, which may perform a regulatory rather than catalytic role within the SnRK1 complex (Baena-González and Hanson 2017). Changes in *AKIN11* expression, which encodes the alpha subunit of the SnRK1 trimer, were also found (Fig. 8). *AKIN11* expression has previously been shown to be altered in Arabidopsis with altered T6P (Schluepmann et al., 2004). Coincident with changes in SnRK1 was significantly less expression of endogenous trehalose pathway genes (Fig. 9). It was found previously that elevated T6P in Arabidopsis caused changes in expression of trehalose pathway genes, particularly class II TPSs (Zhang et al., 2009) in accordance with them being starvation inducible and Suc repressible (Nunes et al., 2013a; Yadav et al., 2014). Here, these genes were induced in pith and induced to a lesser extent in florets. Our data confirm strong overlap and/or strong direct convergence between the SnRK1 and T6P signaling pathways, with the T6P inhibition of SnRK1 activity as a putative mechanism of metabolic reprogramming.

An association between T6P and Suc is well established (Lunn et al., 2006; Nunes et al., 2013a). In this study, decreased T6P in transgenic pith correlated with less Suc in pith, but less T6P in transgenic florets correlated with more Suc. This confirms that the Suc: T6P nexus (Yadav et al., 2014) is tissue dependent. The effect of T6P on the altered distribution and expression of *SWEET* genes implies a broader role of T6P in movement of Suc between tissues.

### Summary

The data in this study support a model wherein decreased T6P down-regulates primary metabolism but up-regulates secondary metabolism in the form of lipid synthesis. Low T6P resulted in different metabolite profiles in pith and floret with evidence that T6P regulates the movement of assimilate from pith to florets. This movement of Suc may create extra demand for Suc from leaves, giving rise to maintenance of high photosynthetic rates for longer. This indicates that T6P can regulate the balance between primary and secondary metabolism, the balance of assimilate accumulation within component parts of reproductive tissues and the activity of photosynthesis. Hence, changes in T6P in sinks can act as a major regulator of whole plant source/sink balance. Overall the changes in metabolite profiles and gene expression patterns in transgenics were similar under well-watered and drought conditions. This similarity indicates that these transgenics are predisposed to cope with drought and yield better under well-watered conditions by maintaining a source-sink balance that favors Suc allocation to florets. Further, the data indicate that low T6P can up-regulate secondary metabolism in the form of lipid synthesis. A strategy to target T6P in this way would improve both crop yields in different environments and produce qualitative changes in secondary metabolic pathways such as lipid biosynthesis.

## METHODS

### Plant Material, Growth Conditions, and Sampling

Two independent transgenic lines (5217 and 5224) of maize (*Zea mays*) expressing the *OSMADS6: TPP1* construct, wild-type line A188, and *OSMADS6: GUS* were grown in a controlled environment (600 µmol m^−2^ s^−1^, 16 h day, 80/70% relative humidity day/night, 27°C/21°C day/night in Rothamsted compost supplemented with full nutrition as in Nuccio et al., 2015). Five different tissue types from female reproductive tissue (florets, shank, pith, node, and fully expanded leaf next to the developing cob) were sampled at 5-d intervals at four time points, 5 d before pollination to 10 d after pollination (labeled as 1, 2, 3, 4; corresponding to −5, 0, 5, 10 d relative to pollination). Five biological replicates from individual plants were taken for each line, time, and tissue. Tissue was sampled during the middle of the photoperiod, snap frozen in liquid N_2_ and stored at −80°C until analysis. Plants were grown under full irrigation, or with drought imposed 5 d before the first sampling point by withholding water until pots reached 65% of the weight before withholding water, as in Nuccio et al. (2015). This level of drought was maintained throughout the sampling period by weighing plants daily. *OSMADS6: GUS* expression cassette activity was assessed by histochemical localization of GUS protein (described in Nuccio et al., 2015).

### RNA seq

Total RNA was extracted from 100 mg ground maize tissue using the Ribopure Kit (Ambion) according to the manufacturer’s instructions. RNA was quantified using a Nanodrop and RNA integrity quality was checked by Agilent RNA 6000 Nano Kit (Agilent Technology) according to the manufacturer’s instructions.

Sequence libraries were prepared at Syngenta and samples were sequenced on a HiSeq2000 by GeneWiz. FastQ sequence files were examined by FastQC software (v0.11.2). Raw reads were used for mapping to the maize reference genome 5a57 (AGP_v2) with gene models 5b.60 (www.maizegdb.org). Tophat (v2.0.12) with bowtie2 (version 2.2.3) was used for read alignment (Trapnell et al., 2009; Langmead et al., 2009). The maximum read mismatches allowed were six. Reads that mapped to more than six places were randomly reported to six mapped locations. Counts were generated from alignment files in BAM format using a custom Python script. Briefly, the method produces counts for gene regions that are defined by a gene feature file (gff3 format) for reads aligning to that region. If a given read aligned to multiple genomic regions defined as genes, up to six regions received one count each. If the read aligned to more than six genomic loci defined as genes, then the read was discarded. This method avoided loss of useful information in cases where reads aligned equally well to different genes. The annotation file used for gene count generation was customized to include sequences for the trait gene *Tpp1* and the phosphomannose isomerase selectable marker gene used in transformation, as well as maize mitochondria and chloroplast sequences. A further step included filtering by raw counts before any normalization. Genes were only kept if they had a counts per million value of 10 or more in at least three different samples that resulted in a total of 22,714 and 22,748 features (genes) reported for the US and DS studies, respectively.

Read quality was analyzed using FastQC (http://www.bioinformatics.babraham.ac.uk/projects/fastqc/). Most of the samples had fairly similar GC content, consistent with the GC content of the maize genome. To correct for possible GC or length bias, data were normalized using the EDASeq R package (Risso et al., 2011). Differential expression analysis and gene ontology (GO) term and pathway enrichment analyses were conducted using the EdgeR package (Robinson et al., 2010).

### GO Term and Pathway Enrichment Analysis

Gene expression analysis from RNAseq was summarized into genes significantly increased or decreased in both transgenic lines compared to wild type in all tissues under well-watered and drought conditions (Figs. 4 and 6; Supplementary Tables S1–S4). Genes were considered to be differentially expressed if results of statistical tests had a false discovery rate of <0.05 and the effect size, measured as absolute value of log2 fold change, was >1. The experimental design resulted in a total of 80 contrasts of transgenic lines compared to wild-type controls across the five tissue types, four time points, and different watering regimes. Initial contrasts compared individual transgenic events to wild-type controls. Roast implemented in the EdgeR package was used for enrichment of GO terms and sets of genes predicted to code for enzymes in biochemical pathways (Wu et al., 2010). Roast uses a gene set test that assigns a *P* value to a set of genes as a unit, which increases statistical power for interpreting results compared with other available permutation tests or more traditional methods such as Fisher’s Exact tests for gene set enrichment. Gene sets of biochemical pathways and GO terms were assembled from data obtained from the Maize Genetics and Genomics Database (www.maizeGDB.org). For pathway data, BioPax formatted files were downloaded from MaizeCyc2.0.1 and parsed to get all gene models likely encoding enzymes in all maize biochemical pathways and reactions in the data set. These data and GO annotations were formatted into files suitable for use with Roast using ad hoc scripts. Data from mRoast, a version of Roast for multiple gene sets, included multiple test corrected *P* values (false discovery rate) as well as directionality of change of the majority of genes within a defined gene set. For Figure 6, data were condensed by first only reporting pathways significantly enriched (false discovery rate of <0.05) in the same direction by both transgenic events in a particular time point and tissue. This was done for both US and DS plants. Secondly, pathways were only reported if they were significantly perturbed in the same direction in both transgenic events in at least two of the four time points tested. These pathway results were further condensed using the pathway ontology in MaizeCyc2.0.1. Individual pathways were grouped together into higher order biochemical pathways using these ontologies. Only pathways included in this ontology were included; all individual reactions enriched in the data set were eliminated. In cases where multiple pathways were present under a given pathway ontology, a single pathway was chosen as most representative of the pathway category.

### Metabolite Analysis

All extraction and analysis were conducted at Metabolon. Samples were extracted and split into equal parts for analysis on GC/mass spectrometry (MS) and LC/tandem (MS/MS) platforms. Proprietary software was used to match ions to an in-house library of standards for metabolite identification and quantitation by peak area integration. LC/MS was based on a Waters ACQUITY UPLC and a Thermo-Finnigan LTQ mass spectrometer consisting of an electrospray ionization source and linear ion-trap mass analyzer. The sample extract was split into two aliquots, dried, then reconstituted in acidic or basic LC-compatible solvents, each of which contained 11 or more injection standards at fixed concentrations. One aliquot was analyzed using acidic positive ion-optimized conditions and the other using basic negative ion-optimized conditions in two independent injections using separate dedicated columns. Extracts reconstituted in acidic conditions were gradient eluted using water and methanol both containing 0.1% formic acid, while the basic extracts, which also used water/methanol, contained 6.5 mm ammonium bicarbonate. The MS analysis alternated between MS and data-dependent MS2 scans using dynamic exclusion. Samples for GC/MS analysis were redried under vacuum desiccation for a minimum of 24 h prior to being derivatized under dried nitrogen using bistrimethyl-silyl-triflouroacetamide. The GC column was 5% phenyl and the temperature ramp 40°C to 300°C over 16 min. Samples were analyzed on a Thermo-Finnigan Trace DSQ fast-scanning single-quadrupole mass spectrometer using electron impact ionization. The instrument was tuned and calibrated for mass resolution and mass accuracy daily. The information output from the raw data files was automatically extracted as discussed below. Accurate mass determination (LC/MS) and MS/MS fragmentation (LC/MS/MS) for structural elucidation was based on a Waters ACQUITY UPLC and a Thermo-Finnigan OrbiElite mass spectrometer, which had a linear ion-trap front end and an orbitrap mass spectrometer back end. Accurate mass measurements could be made on the parent ion as well as fragments. The typical mass error was <5 ppm.

The informatics system consisted of four major components: the Laboratory Information Management System, the data extraction and peak-identification software, data processing tools for QC and compound identification, and a collection of information interpretation and visualization tools for use by data analysts. The hardware and software foundations for these informatics components were the LAN backbone and a database server running Oracle 10.2.0.1 Enterprise Edition. Peaks were identified using Metabolon’s proprietary peak integration software, and component parts were stored in a separate and specifically designed complex data structure. Compounds were identified by comparison to library entries of purified standards or recurrent unknown entities.

For statistical analysis, pairwise comparisons using Welch’s T-tests and/or Wilcoxon’s rank sum tests were performed. ANOVA was also conducted. Metabosync (Metabolon Inc.) was used to show changes in metabolite abundance in representative contrasts of transgenic event 5,224 compared to wild type grown in US conditions to show the full extent of metabolite changes using Welch’s T-tests to calculate the size of the circle and statistical significance of difference of mean values (Fig. 5, A and B).

### SnRK1 Activity

Total soluble protein was extracted from 200 mg of tissue ground under liquid nitrogen in a pestle and mortar in 600 µL of ice-cold homogenization buffer of 100 mm tricine-NaOH, pH 8, 25 mm NaF, 5 mm dithiothreitol, 2 mm tetrasodium pyrophosphate, 0.5 mm EDTA, 0.5 mm EGTA, 1 mm benzamidine, 1 mm phenylmethylsulfonyl fluoride, 1 mm protease inhibitor cocktail (Sigma P9599), phosphatase inhibitors (PhosStop; Roche), and insoluble polyvinylpyrrolidone to 2% (w/v). Homogenate was centrifuged at 13,000*g* at 4°C. Supernatant (250 µL) was desalted in illustra NAP-5 columns (GE Healthcare) pre-equilibrated with homogenization buffer. Eluent was supplemented with protease inhibitor cocktail and okadaic acid to 2.5 mm before freezing in liquid nitrogen. SnRK1 activity of three replicates for each time point was determined as described by Zhang et al. (2009) in a final volume of 25 µL in microtitre plate wells at 30°C. Assay medium was 40 mm HEPES-NaOH, pH 7.5, 5 mm MgCl_2_, 200 mm ATP containing 12.5 kBq [γ-^33^P] ATP (PerkinElmer), 200 µm AMARA peptide (Enzo Life Sciences), 5 mm dithiothreitol, 1 µm okadaic acid, and 1 mm protease inhibitor cocktail (Sigma P9599). Assays were started with 5 µL extract and stopped after 6 min by transferring 15 µL to 4 cm^2^ squares of Whatman P81 phosphocellulose paper immersed immediately in 1% phosphoric acid. These were then washed with four 800-mL volumes of 1% phosphoric acid, immersed in acetone for 15 min, air dried, and transferred to vials with 3.5 mL of scintillation cocktail (Ultima Gold).

### Photosynthesis

Leaf gas exchange measurements of well-watered and water-stressed maize plants were carried out using a portable infra-red open gas exchange system (LI-6400XT; LI-COR) under the growing conditions (ambient CO_2_, 400 µL L^–1^), leaf temperature 27°C, photosynthetic photon flux density 600 µmol m^–2^ s^–1^, and relative air humidity 65 ± 5%. Each leaf reached a steady state of CO_2_ uptake in the leaf chamber before measurements were taken.

### Accession Numbers

Maize SnRK1 gene accession numbers: SnRK1beta, GRMZM2G025459; AKINB, GRMZM2G138814; AKIN10B, GRMZM2G077278; AKIN10, GRMZM2G077278; AKIN11, GRMZM2G107867.

Maize trehalose pathway gene accession numbers: TPPA1, GRMZM2G178546; TPPB1.3, GRMZM2G174396; TPPA.3, GRMZM2G112830; TPPB.1.2, GRMZM2G140078; TPPB.1.4, GRMZM2G055150; TPSII.3.2, GRMZM2G123277; TPSII.4.2, GRMZM2G008226; TPSII.4.1GRMZM2G527891; TPSII.2.1, GRMZM2G019183; TPSII.5.4, GRMZM2G122231; TPSII.4.3, GRMZM2G366659; TPSII.5.3, GRMZM2G312521; TPPB.2.1, GRMZM2G014729.

Maize SWEET gene accession numbers: SWEET1b, GRMZM2G153358; SWEET2, GRMZM2G324903; SWEET3a, GRMZM2G179679; SWEET11, GRMZM2G368827; SWEET13a, GRMZM2G173669; SWEET13c, GRMZM2G179349; SWEET14b, GRMZM2G015976; SWEET15a, GRMZM2G168365; SWEET15b, GRMZM5G972392; SWEET16, GRMZM2G107597.

Maize allantoinase gene accession number: GRMZM2G173413.

RNAseq accession: PRJNA421180 ID: 421180; US samples: SUB3315967; DS samples: SUB3357197 https://www.ncbi.nlm.nih.gov/bioproject/PRJNA421180

### Supplemental Data

The following supplemental materials are available.

Supplemental Figure S1. Histochemical analysis of *OSMADS6: GUS*.
Supplemental Figure S2. Histochemical analysis of *OSMADS6: GUS* activity in node.
Supplemental Figure S3. Effect of *OSMADS6: TPP1* on selected metabolites in reproductive tissues and leaves during early reproductive development.
Supplemental Table S1. Changes in gene expression.
Supplemental Table S2. Changes in gene expression.
Supplemental Table S3. Changes in gene expression.
Supplemental Table S4. Changes in gene expression.
Supplemental Table S5. SnRK1 marker gene expression previously identified in Baena-González et al. (2007); Zhang et al. (2009); Martinez-Barajas et al. (2011) in transgenics compared to wild type.
Supplemental Table S6. SnRK1 gene expression in transgenics compared to wild type.
Supplemental Table S7. Trehalose pathway gene expression in transgenics compared to wild type.
Supplemental Table S8. SWEET gene expression in transgenics compared to wild type.

## Supplementary Material

Supplementary Data
