# Supplementary material for: Trehalose 6-Phosphate Regulates Photosynthesis and Assimilate Partitioning in Reproductive Tissue
Source: Plant Physiol. 2018 Feb 6;176(4):2623–38. doi: 10.1104/pp.17.01673 (PMC5884609; doi:10.1104/pp.17.01673)
Supplement: Supplementary Data [file plphys_v176_4_2623_s1.zip › PP2017-RA-01673R2_Supplemental_Materialfigs.pdf]

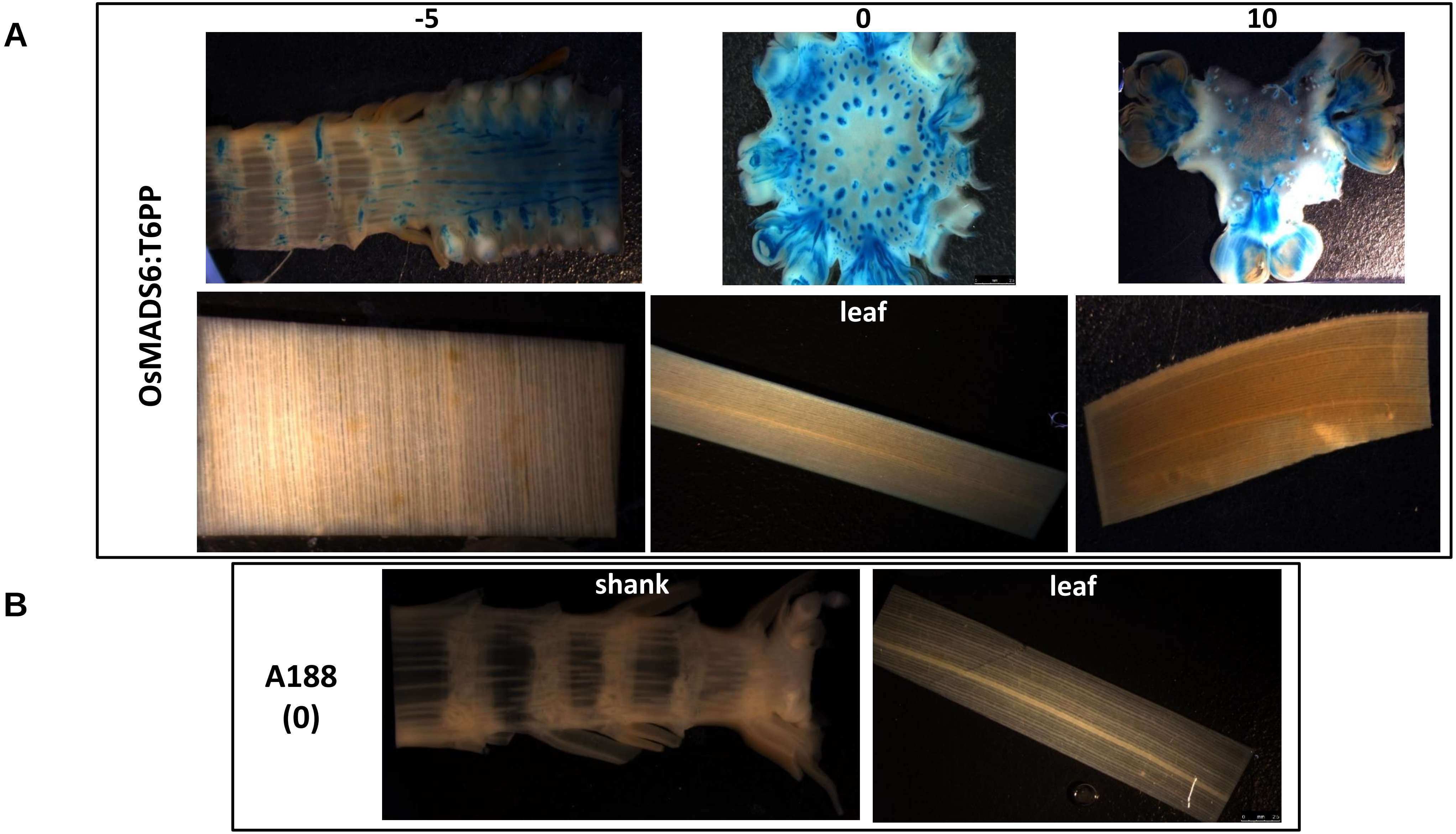

Supplementary Figure S1. Histochemical evaluation of GUS activity in select maize tissues. At the indicated time points tissues from (A) plants containing the *OSMADS6: GUS* transgene and (B) wildtype controls were harvested and subject to histochemical analysis. Tissues were incubated in the histochemical reagent for 12 hours then cleared with ethanol. Several samples were analyzed for each tissue, a representative image is shown. The time points are 5 days before pollination (-5), day of pollination (0), 5 days after pollination (5), and 10 days after pollination (10).

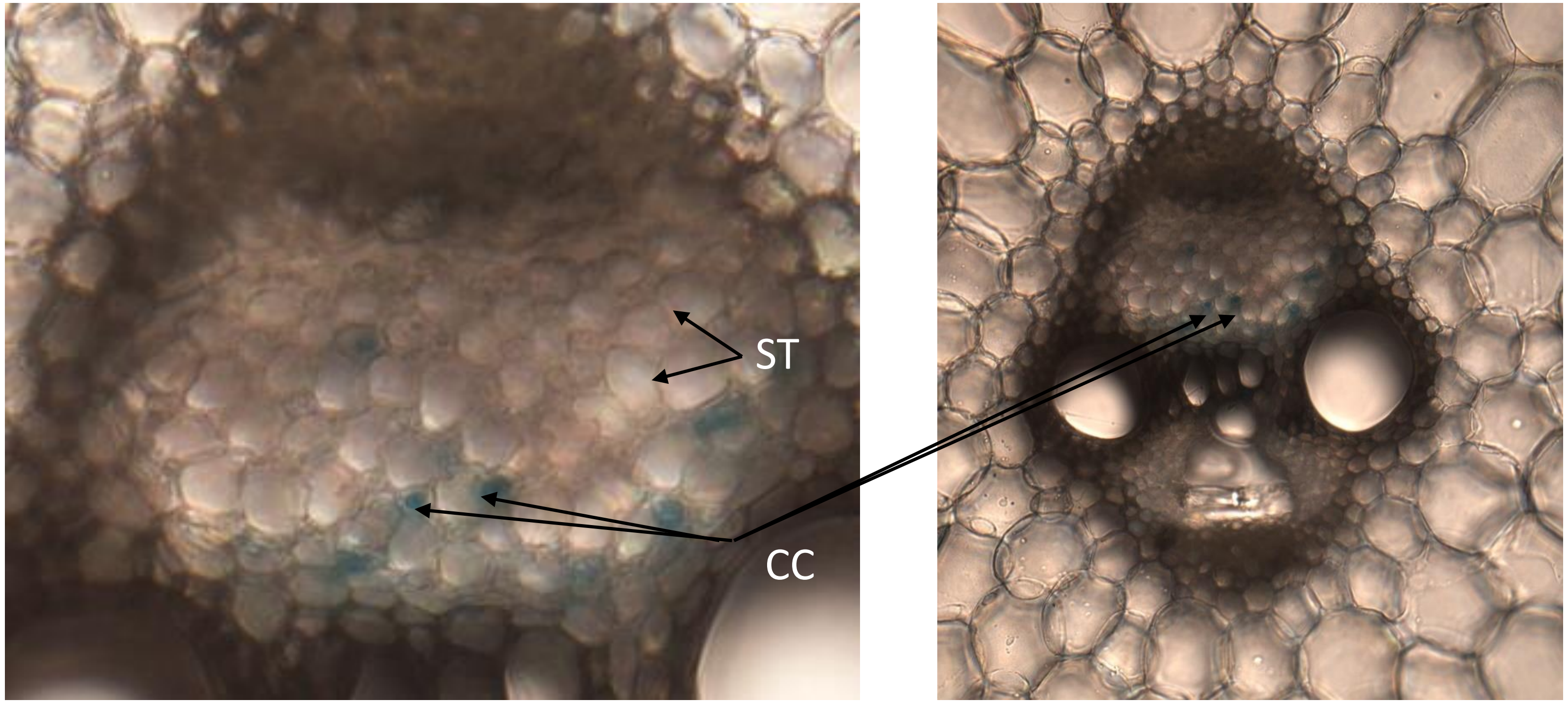

Supplementary Figure S2. Histochemical analysis of *OSMADS6: GUS* activity in node. High resolution image of a representative vascular bundle shown in Figure 2O. Sieve tube (ST) elements and companion cells (CC) are indicated by the black arrows. Pictures are from two independent transgenic events.

Supplementary Figure S3. Effect of the OsMads6-Tpp1 gene on metabolites in floret, node, pith, shank and leaf during early reproductive development. The heat map shows the ratio of the transgenic event (5217, 5224) to wildtype signal in **(A)** unstressed plants. Red and green shaded cells indicate the OsMads6:Tpp1 values are significantly higher and lower, respectively, than wildtype values ($P \leq 0.05$). Light red and light green shaded cells indicate OsMads6:Tpp1 values that trend higher and lower, respectively, than wildtype ($0.05 < P < 0.10$). Data represent 5 biological reps. The time points are 5 days before pollination (-5), day of pollination (0), 5 days after pollination (5), and 10 days after pollination (10).

Floret (Days relative to pollination)

Biochemical	5217				5224			
	-5	0	5	10	-5	0	5	10
sucrose	1.15	1.11	0.93	1.08	1.1	1.16	0.92	1.22
glucose	0.95	1.13	1.19	1.33	0.98	1.05	1.19	1.26
fructose	0.95	0.95	1.13	1.19	1.01	0.99	1.01	1.18
raffinose	1.37	1	1.75	0.92	1.5	1.09	1.95	1.38
xylose	1.04	0.91	0.78	1	1.1	0.73	0.63	0.84
xylulose	0.94	0.96	0.97	1.35	1.04	1.08	0.85	1.33
glucose-6-phosphate (G6P)	1.28	1.37	0.76	1.31	1.57	1.39	0.77	1.69
fructose-6-phosphate	1.47	1.22	0.96	1.35	2.04	1.24	0.89	1.42
alpha-ketoglutarate	0.65	1.02	0.73	1.05	0.68	0.84	0.77	1.95
citrate	1.3	1.14	1.13	1.19	1.3	0.98	1.13	1.19
inositol 1-phosphate (I1P)	1.1	1.33	0.75	0.9	1.13	1.19	0.82	1.07
myo-inositol	1.1	1.56	1.33	1.18	1.01	1.45	1.45	1.26
adenine	0.97	0.85	0.8	0.91	0.92	0.77	0.69	0.87
adenosine	0.88	1.06	0.98	0.97	0.84	1.04	0.92	0.86
adenosine 5'-monophosphate (AMP)	1.81	1.31	0.99	1.23	2.11	1.51	0.88	1.65
guanosine	0.88	1.08	1.05	1.04	0.85	1.05	0.96	1.05
allantoin	2.58	2.91	1.08	0.96	2.57	2.41	1.16	0.71
uridine	0.93	0.98	0.94	0.93	0.88	0.96	0.93	0.88
cysteine	1.28	1.79	1.15	0.78	1.12	1.55	0.83	0.76
glycine	2.24	2.11	1.14	1.1	1.64	1.46	0.76	1.05
serine	1.19	1.56	1.05	0.94	1.4	1.43	0.88	0.85
S-methylmethionine	1.39	1.21	0.84	0.88	1.5	1.26	0.9	0.95
phenylalanine	1.02	1.5	1.15	1.11	1.11	1.65	1.36	0.94
tyrosine	1.03	1.24	1.16	1.14	1.11	1.31	1.13	1.05
alanine	1.87	2.11	0.96	0.97	1.88	1.83	0.79	1.02
asparagine	2.73	1.77	0.81	0.82	2.41	1.35	0.69	0.65
aspartate	1.11	1.28	0.88	0.9	1.22	1.09	0.74	0.76
homoserine	1.89	1.75	1.36	1.59	1.29	1.22	1.08	1.1
lysine	1.19	1.29	0.69	0.77	1.42	1.48	0.8	0.87
methionine	1.15	1.15	1	1.13	1.19	1.11	0.94	1.06
arginine	1.23	1.19	0.75	0.68	1.83	1.26	0.87	0.8
glutamate	1.01	1.03	1	0.97	1.11	1.13	1.09	1.34
glutamine	1.14	1.14	1.08	1	1.14	1.1	1.05	0.96
histidine	1.45	1.32	0.68	0.74	1.37	1.36	0.68	0.82
proline	1.24	1.11	1.19	1.46	1.58	1.1	1.14	1.42
leucine	1.2	1.32	0.95	0.96	1.24	1.35	0.96	0.81
valine	1.32	1.31	1.06	1.06	1.4	1.31	1.06	0.97
glutathione, oxidized (GSSG)	3.21	1.39	0.71	1.29	3.59	1.2	0.49	0.33
glutathione, reduced (GSH)	3.45	1.11	1.42	1.25	5.98	1.19	1.19	1.2
1-oleoylglycerophosphoinositol*	1.22	1.21	1.87	1.94	1.39	1.31	1.66	2.16
1-palmitoylglycerophosphate	0.97	1.28	1.59	1.5	1.24	1.31	1.57	1.23
1-palmitoylglycerophosphocholine (16:0)	1.4	1.41	1.66	1.48	2.82	1.79	1.79	1.81
1-palmitoylglycerophosphoethanolamine	0.92	1.4	1.62	1.13	1.12	1.74	1.55	1.32
1-palmitoylglycerophosphoinositol*	0.85	1.44	2.03	1.99	1.26	1.53	1.97	1.9
1-stearoylglycerophosphocholine (18:0)	0.69	1.47	1.9	1.27	1.67	1.59	2.05	1.14
2-palmitoylglycerophosphocholine*	1.06	1.37	1.77	1.86	1.74	1.95	1.53	1.77
glycerol 3-phosphate (G3P)	1.21	1.35	0.89	0.89	1.58	1.33	0.8	0.86
phosphoethanolamine	1.47	1.33	0.49	0.58	1.4	1.06	0.41	0.58
phytosphingosine	1.47	1.35	1.22	1.4	1.67	1.14	1.12	1.21
sphinganine	1.55	1.59	1.78	1.2	1.78	1.52	1.94	1.11
beta-sitosterol	1.22	1.27	1.11	1.04	1.21	1.21	1.14	0.96

Node (Days relative to pollination)

Biochemical	5217				5224			
	-5	0	5	10	-5	0	5	10
sucrose	1.03	1.24	1.29	1.12	0.96	1.29	1.11	1.03
glucose	1.13	0.94	1.13	1.05	1.12	0.95	1.25	1.17
fructose	1.12	1.07	1.19	1.14	1.13	1.1	1.26	1.21
raffinose	0.38	0.6	0.62	0.54	1.25	1.19	1.12	1.15
xylose	0.95	0.97	1.14	1.04	1.07	0.94	1.28	1.06
xylulose	0.47	1.14	0.72	1.27	1.18	1.15	0.78	1.19
glucose-6-phosphate (G6P)	1.09	0.98	0.95	0.83	1.15	1.02	1.01	0.9
fructose-6-phosphate	0.92	0.87	0.97	0.84	0.95	0.97	1.11	0.83
alpha-ketoglutarate	1.13	1.24	1.43	0.87	0.73	0.8	1.12	0.82
citrate	1.29	1.03	1.09	0.9	0.96	0.67	0.85	0.71
inositol 1-phosphate (I1P)	1.08	1.01	1.07	0.78	1.27	1.39	1.18	0.93
myo-inositol	1.15	1.25	1.2	1.16	1	0.94	1.07	0.95
adenine	1.22	1.09	0.97	0.79	0.77	1	0.69	0.89
adenosine	1.06	1.04	1.01	0.99	0.93	0.95	0.9	0.92
adenosine 5'-monophosphate (AMP)	0.84	0.7	0.97	1.05	0.82	0.68	0.87	0.67
guanosine	1.16	0.98	0.99	0.95	0.94	0.88	0.88	0.89
allantoin	1.23	1.39	1.28	1.09	1.36	1.36	1.23	1.07
uridine	1.06	1.11	0.97	0.98	0.97	1	0.88	0.93
cysteine	1.83	0.96	1.27	0.79	1.97	0.89	0.88	0.85
glycine	0.88	0.56	0.76	0.69	0.87	0.49	0.56	0.54
serine	1.04	0.74	0.82	0.69	1.09	0.69	0.77	0.73
S-methylmethionine	0.99	0.86	0.68	0.82	0.81	0.68	0.69	0.66
phenylalanine	0.99	0.78	0.81	0.96	1.08	0.83	0.89	0.92
tyrosine	1.01	0.98	1.01	1.09	0.95	0.93	0.9	0.9
alanine	0.82	0.61	0.64	0.8	1.04	0.7	0.75	0.87
asparagine	1.06	0.61	0.65	0.6	1.18	0.4	0.65	0.57
aspartate	0.99	0.84	0.78	0.66	0.87	0.61	0.81	0.5
homoserine	2.17	0.57	1.63	0.91	1.11	0.49	0.81	0.5
lysine	1	0.96	0.98	0.93	0.9	0.87	0.94	0.94
methionine	1.05	1.03	0.92	0.89	0.88	0.88	0.75	0.74
arginine	1.02	0.89	0.81	0.76	0.89	0.7	0.78	0.82
glutamate	0.99	0.86	0.86	0.9	1.11	0.89	1.25	0.93
glutamine	1.19	1.06	1.12	0.91	0.85	0.74	0.69	0.75
histidine	0.85	0.76	0.8	0.88	0.87	0.74	0.8	0.77
proline	1	0.74	0.86	1	1.23	0.8	1.27	1.13
leucine	0.93	0.6	0.67	0.83	1.02	0.61	0.88	0.86
valine	1	0.74	0.89	0.95	1.03	0.75	0.88	0.93
glutathione, oxidized (GSSG)	0.87	0.73	0.99	0.85	0.92	0.64	1.17	1
glutathione, reduced (GSH)	0.73	0.7	0.99	0.96	0.89	0.64	0.84	0.81
1-oleoylglycerophosphoinositol*	1	1	1	1	1	1	1	1
1-palmitoylglycerophosphate	1.08	1.04	0.94	1.05	1.22	0.98	0.76	0.89
1-palmitoylglycerophosphocholine (16:0)	1.24	0.79	1.49	1.05	0.81	0.72	1	0.98
1-palmitoylglycerophosphoethanolamine	0.88	0.9	1.19	0.89	0.76	1.1	1.19	0.79
1-palmitoylglycerophosphoinositol*	0.96	0.96	1.3	1.21	0.71	0.82	0.96	0.93
1-stearoylglycerophosphocholine (18:0)								
2-palmitoylglycerophosphocholine*	1	1	1	1	1	1	1	1
glycerol 3-phosphate (G3P)	1.01	1.03	1	0.93	0.95	0.85	0.97	0.79
phosphoethanolamine	1.43	0.52	0.75	1.73	1.89	0.43	0.75	1
phytosphingosine	1.22	0.85	1.84	1.5	1.09	0.94	1.32	1.13
sphinganine	1.35	1.39	2.46	0.75	1.1	1.28	1.4	0.71
beta-sitosterol	1.11	1.05	1.15	1.27	0.97	1.11	1	1.02

Pith (Days relative to pollination)

Biochemical	5217				5224			
	-5	0	5	10	-5	0	5	10
sucrose	1.17	0.71	0.65	0.88	1.18	1.32	0.46	0.77
glucose	1.08	1	1.12	1.15	1.07	1.07	1.12	1.33
fructose	1.03	1.12	0.83	1.03	1.01	1.19	0.98	1.18
raffinose	0.69	1	1.37	0.8	0.69	1	2.33	1.25
xylose	1.04	0.72	0.68	0.75	0.89	0.78	0.5	0.61
xylulose	0.73	0.7	0.88	0.76	0.43	0.72	0.48	1.11
glucose-6-phosphate (G6P)	0.87	1.08	0.81	0.75	1.24	1.51	0.37	0.54
fructose-6-phosphate	0.75	0.74	0.5	0.69	0.9	0.77	0.35	0.42
alpha-ketoglutarate	0.58	0.75	0.68	0.48	0.4	0.73	0.45	0.42
citrate	1.05	2.07	1.02	1.34	1.33	1.98	1.05	1.42
inositol 1-phosphate (I1P)	1.15	0.89	0.6	0.52	1.03	0.79	0.35	0.44
myo-inositol	0.93	2.31	1.87	1.19	1.06	2.33	2.17	0.99
adenine	1.11	0.82	0.87	0.83	0.91	0.82	0.79	0.84
adenosine	0.87	0.89	0.71	0.87	0.82	0.86	0.46	0.7
adenosine 5'-monophosphate (AMP)	0.47	0.87	0.42	0.39	0.67	0.84	0.3	0.48
guanosine	0.83	0.83	0.67	0.74	0.85	0.74	0.45	0.69
allantoin	1.14	1.59	1.62	0.9	1.28	1.46	1.43	0.52
uridine	0.88	0.85	0.68	0.68	0.83	0.81	0.51	0.71
cysteine	0.89	1.08	0.73	0.5	0.26	0.7	0.45	0.36
glycine	1.32	0.82	0.23	0.3	0.85	0.68	0.13	0.44
serine	0.84	1.07	0.41	0.42	1.18	1.11	0.27	0.44
S-methylmethionine	0.65	0.66	0.22	0.18	0.79	0.78	0.27	0.25
phenylalanine	0.75	1.75	0.83	0.85	1.02	1.97	0.43	0.65
tyrosine	0.89	0.94	0.42	0.56	0.91	0.8	0.33	0.55
alanine	1.05	1.2	0.56	0.61	1.21	1.16	0.48	0.94
asparagine	1.08	1.04	0.2	0.08	1.31	0.66	0.03	0.11
aspartate	0.84	1.06	0.44	0.56	0.96	1.08	0.31	0.38
homoserine	2.18	1.41	0.36	1	1.23	0.6	0.36	1.57
lysine	0.72	0.56	0.5	0.49	0.86	0.96	0.48	0.43
methionine	0.88	0.96	0.56	0.52	0.95	0.89	0.45	0.51
arginine	0.66	0.41	0.18	0.12	0.89	0.8	0.21	0.11
glutamate	0.92	0.97	0.46	0.52	0.98	1.11	0.31	0.45
glutamine	0.99	0.98	0.38	0.28	0.92	0.95	0.29	0.36
histidine	0.84	0.56	0.42	0.29	0.88	0.6	0.27	0.3
proline	0.91	1.06	0.69	0.94	1.17	0.97	0.74	1.6
leucine	0.87	1.07	0.52	0.35	0.95	1.17	0.21	0.24
valine	1.01	1.17	0.49	0.41	1.19	1.2	0.28	0.48
glutathione, oxidized (GSSG)	1.06	1.11	0.36	0.49	0.94	1.05	0.43	0.35
glutathione, reduced (GSH)	0.86	1.11	0.62	0.71	1.13	0.95	0.46	0.48
1-oleoylglycerophosphoinositol*	0.92	1	1	1	0.81	1	1	1
1-palmitoylglycerophosphate	0.99	1.47	0.98	1.05	0.78	1.03	1	0.71
1-palmitoylglycerophosphocholine (16:0)	1.54	1.68	0.99	0.39	1.32	1.47	1.53	0.88
1-palmitoylglycerophosphoethanolamine	0.98	1.34	1	0.62	1.11	1.33	1.36	0.94
1-palmitoylglycerophosphoinositol*	1.27	1.42	0.86	0.66	0.91	1.15	1.29	0.57
1-stearoylglycerophosphocholine (18:0)								
2-palmitoylglycerophosphocholine*	1.22	1	1	1	0.82	1	1	1
glycerol 3-phosphate (G3P)	0.92	0.98	0.68	0.74	0.99	1.2	0.6	0.59
phosphoethanolamine	0.53	1.06	0.28	0.32	0.78	1.04	0.13	0.32
phytosphingosine	0.91	0.62	1.33	0.99	0.88	0.9	1.37	0.96
sphinganine	1.2	1.45	1.07	1.13	1.19	1.19	1.05	1
beta-sitosterol	0.95	1.17	0.85	1.18	0.99	1.38	0.92	1.28

Shank (Days relative to pollination)

Biochemical	5217				5224			
	-5	0	5	10	-5	0	5	10
sucrose	0.96	0.7	1.12	1.11	0.92	0.74	1.07	1.11
glucose	0.92	0.94	1.01	0.99	0.9	1	0.93	0.93
fructose	1	0.94	1.08	1.09	1.04	1.06	1.06	1.08
raffinose	0.8	0.6	0.91	0.3	0.76	0.7	0.91	0.35
xylose	0.94	0.98	1.2	0.95	0.9	1.02	1.22	0.88
xylulose	1.39	0.87	1.22	1.19	1.89	0.61	1.24	1.8
glucose-6-phosphate (G6P)	1.05	0.9	1.01	0.77	1.03	0.77	0.85	0.64
fructose-6-phosphate	1	1.06	0.68	0.6	1.08	1.28	0.61	0.62
alpha-ketoglutarate	0.9	0.7	1.14	1.15	0.81	0.63	1.09	0.91
citrate	1.32	0.96	1.23	0.91	1.05	0.88	1.06	0.82
inositol 1-phosphate (I1P)	0.87	0.78	0.89	0.84	0.89	0.79	0.95	0.73
myo-inositol	0.83	0.79	1.18	1.01	0.71	0.81	1.06	0.77
adenine	0.86	0.9	0.95	0.75	0.87	0.9	0.92	0.95
adenosine	0.99	0.83	1.06	0.95	1.02	0.81	1.11	0.77
adenosine 5'-monophosphate (AMP)	1.1	0.92	1.23	1.02	1.14	0.85	0.54	0.45
guanosine	1.05	0.9	0.98	0.95	1.1	0.83	1.03	0.82
allantoin	1.13	1.34	1.44	1.23	1.3	1.57	1.62	1.15
uridine	1.1	1.01	0.91	0.92	1.09	0.94	0.99	0.81
cysteine	1.13	0.91	1.23	0.76	1.11	0.87	1.19	0.62
glycine	1.11	1.12	0.99	0.63	1.1	1.03	1.15	0.54
serine	0.91	0.79	1.08	0.65	1.05	0.94	1.27	0.63
S-methylmethionine	1.05	1	1.35	1.13	1.43	1.19	1.3	0.84
phenylalanine	0.93	0.86	1.05	0.85	1.06	0.91	1.13	0.65
tyrosine	1.05	0.94	1.15	0.89	1.04	0.93	1.14	0.73
alanine	0.98	0.89	1.15	0.65	1.06	1.1	1.17	0.69
asparagine	1.19	1.28	1.44	0.72	1.55	1.36	1.74	0.64
aspartate	1.04	0.82	1.39	0.63	1.08	0.92	1.47	0.58
homoserine	1.46	1.36	2.79	1.13	0.95	1.11	2.83	0.53
lysine	1.08	0.93	1.69	1.32	1.2	1.02	1.54	0.9
methionine	1.14	0.9	1.03	0.87	1.06	0.87	1	0.8
arginine	0.9	1.06	1.67	1.43	1.48	1.18	1.59	1.01
glutamate	1.1	1.02	1.37	0.8	1.22	1.29	1.42	1.01
glutamine	1.17	1.09	1.11	0.94	1.2	1.06	0.93	0.92
histidine	1.02	1.01	1.32	0.92	1.18	1.21	1.5	0.77
proline	1.01	0.92	0.98	1.19	1.12	0.96	1.97	1.35
leucine	1.06	0.85	1.21	0.8	1.24	0.98	1.55	0.67
valine	1.11	1.01	1.15	0.8	1.24	1.05	1.31	0.7
glutathione, oxidized (GSSG)	2.69	0.97	1.22	0.93	2.54	0.92	1.13	0.54
glutathione, reduced (GSH)	1.5	1.13	1.21	0.97	1.47	1.03	0.95	0.94
1-oleoylglycerophosphoinositol*	1.02	0.8	1	1	1.45	0.93	1	1
1-palmitoylglycerophosphate	0.8	0.99	0.86	0.83	0.88	0.98	1.1	1.02
1-palmitoylglycerophosphocholine (16:0)	1.2	1.16	0.88	2.41	1.39	1.24	1.19	1.9
1-palmitoylglycerophosphoethanolamine	0.79	1.01	0.92	1.1	1	1.06	1.23	1.61
1-palmitoylglycerophosphoinositol*	0.9	1.05	0.81	1.86	1	1.07	1.38	1.93
1-stearoylglycerophosphocholine (18:0)								
2-palmitoylglycerophosphocholine*	0.72	0.91	1.3	1	0.98	1.05	1.79	1
glycerol 3-phosphate (G3P)	0.9	0.91	1.14	0.88	1.05	1	1.04	0.66
phosphoethanolamine	0.67	0.86	1.47	0.64	0.93	0.83	1.26	0.36
phytosphingosine	2.23	1.03	0.83	0.99	2.56	0.99	0.57	0.72
sphinganine	2.36	1.07	1.25	0.88	2.34	0.96	0.72	0.45
beta-sitosterol	0.95	0.87	1.08	0.85	0.94	0.87	0.99	0.89

Leaf (Days relative to pollination)

Biochemical	5217				5224			
	-5	0	5	10	-5	0	5	10
sucrose	1.22	1.19	1.17	1.19	1.2	1.18	1.23	1.09
glucose	1.07	0.96	0.75	1.24	1.09	1.05	0.8	1.11
fructose	1.14	1.04	0.7	1.21	1.11	1.11	0.78	1.06
raffinose	1.47	1.33	1.06	1.27	1.63	1.16	1.05	0.96
xylose	1.6	1.35	1.01	1.35	1.01	1.44	0.82	1.07
xylulose								
glucose-6-phosphate (G6P)	1.02	0.89	0.86	1.21	1.06	1.21	0.93	1.19
fructose-6-phosphate	0.93	0.9	0.79	0.99	0.95	1.21	0.87	1.08
alpha-ketoglutarate	1.15	1.55	1.06	1.19	0.8	1.44	0.87	0.87
citrate	1.13	1.13	0.73	1.09	0.9	1.13	0.67	1.08
inositol 1-phosphate (I1P)	1.05	0.85	0.77	1.29	0.88	1.31	0.67	1.14
myo-inositol	1.16	1.13	0.94	1.2	1.11	1.26	0.95	1.13
adenine	0.84	0.67	0.79	0.68	0.6	0.66	0.72	0.72
adenosine	1.09	1.11	1.02	1.19	1.18	1.12	1.02	1.03
adenosine 5'-monophosphate (AMP)	1.07	1.15	0.91	0.61	1.38	1.48	0.77	0.83
guanosine	1.17	1.24	1.02	1.21	1.3	1.16	0.98	1.1
allantoin	4.93	3.13	9.18	5.41	1.77	2.26	4.52	5.66
uridine	1.07	1.08	1.04	1.14	1.11	1.01	0.96	1.01
cysteine	1.37	1.13	1.03	0.94	1.07	1.1	1.03	1.07
glycine	2.31	1.29	1.36	1.31	0.98	0.92	1.02	1.02
serine	1.17	0.95	1.94	0.46	1.11	1.02	1.88	0.63
S-methylmethionine								
phenylalanine	0.95	1.01	0.76	0.91	1.06	0.94	0.86	0.75
tyrosine	1.01	1.05	0.75	1.03	1.12	1.09	0.8	0.79
alanine	1.7	1.33	1.29	1.48	1.09	1.23	1.01	1.33
asparagine	1.58	0.86	1.4	0.75	1.08	0.77	1.38	2.23
aspartate	1.06	1.09	1.11	0.6	1.07	1.18	0.78	1.08
homoserine	1.35	1.52	1.32	0.99	0.85	1.46	1.02	1.17
lysine	1.12	1.2	0.83	1.17	1.04	1.2	0.82	1.2
methionine								
arginine								
glutamate	1.09	1.04	1.26	1.01	0.86	0.96	0.88	0.91
glutamine	1.13	0.97	1.29	0.84	1.12	1.03	0.99	1.15
histidine								
proline	0.94	0.86	0.78	1.16	1.14	0.79	1.35	0.9
leucine	0.86	1.02	0.65	0.88	1	0.98	0.81	0.74
valine	1.14	1.05	0.96	1.07	1.07	0.99	0.94	0.9
glutathione, oxidized (GSSG)								
glutathione, reduced (GSH)								
1-oleoylglycerophosphoinositol*								
1-palmitoylglycerophosphate	1.14	0.85	0.62	0.99	1.08	0.81	0.91	0.86
1-palmitoylglycerophosphocholine (16:0)	1.05	0.83	0.87	1.9	0.94	1.08	1.32	1.53
1-palmitoylglycerophosphoethanolamine								
1-palmitoylglycerophosphoinositol*	1.1	0.79	0.46	1.32	1.41	0.97	0.82	0.98
1-stearoylglycerophosphocholine (18:0)								
2-palmitoylglycerophosphocholine*								
glycerol 3-phosphate (G3P)	1.1	1.24	0.98	1.58	1.06	1.16	1.09	1.32
phosphoethanolamine								
phytosphingosine	0.94	0.82	0.59	1.12	0.99	1.03	0.75	1.03
sphinganine								
beta-sitosterol	1.05	1.2	0.83	1.08	1.11	1.43	1.08	1.1

Supplementary Figure S3. Effect of OsMads6-Tpp1 on metabolites in floret, node, pith, shank and leaf during early reproductive development. The heat map shows the ratio of the transgenic event (5217, 5224) to wildtype signal in **(B)** drought stressed plants**.** Red and green shaded cells indicate the OsMads6:Tpp1 values are significantly higher and lower, respectively, than wildtype values ($P \leq 0.05$). Light red and light green shaded cells indicate OsMads6:Tpp1 values that trend higher and lower, respectively, than wildtype ($0.05 < P < 0.10$). Data represent 5 biological reps. The time points are 5 days before pollination (-5), day of pollination (0), 5 days after pollination (5), and 10 days after pollination (10).

Biochemical	Floret								Node								Pith								Shank								Leaf							
	5217				5224				5217				5224				5217				5224				5217				5224				5217				5224			
	Days relative to pollination								Days relative to pollination								Days relative to pollination								Days relative to pollination								Days relative to pollination							
	-5	0	5	10	-5	0	5	10	-5	0	5	10	-5	0	5	10	-5	0	5	10	-5	0	5	10	-5	0	5	10	-5	0	5	10	-5	0	5	10	-5	0	5	10
sucrose	1.22	1.23	0.85	0.81	1.22	1.21	1.12	1	1.11	1.21	1.06	1.06	0.92	0.99	0.96	0.93	0.7	1.02	0.43	0.81	1.17	1.57	0.43	0.78	0.71	0.42	1.05	1.03	0.84	0.73	1.09	0.96	1.17	1.42	1.26	1.42	1.22	1.24	1.45	1.32
glucose	1.12	1.04	1.18	1.15	1.1	1.07	1.24	1.21	1.14	1.03	1.06	1.5	1.03	1.06	0.96	1.24	1.04	1.12	1.33	1.16	1.09	1.1	1.58	1.37	1.07	1.04	1.05	1.02	0.99	1.03	1.13	1.19	0.91	0.86	1.03	1.32	1	0.91	1.53	1.76
fructose	1.1	1.01	1.22	1.15	1.09	1.03	1.28	1.26	1.15	1.08	1.05	1.66	1.1	1.15	0.95	1.41	1.01	1.08	1.42	1.28	1.04	1.06	1.59	1.48	1.07	1.01	1.07	1.05	0.99	1.06	1.16	1.24	0.92	0.91	1.01	1.35	0.96	0.94	1.38	1.65
raffinose	3.19	0.47	4.4	0.5	1.67	0.78	2.38	1.58	0.77	0.74	0.72	0.57	0.35	1.62	1.38	1.3	1.15	1.06	1.04	0.87	5.2	0.44	0.98	0.96	1.22	0.43	1.16	0.9	0.94	0.95	1.33	1.03	1.48	0.99	2.2	0.96	1.21	1.25	1.31	0.92
xylose	1.01	0.84	0.82	1.25	1.34	0.85	0.67	1.05	1.09	1.14	0.94	1.29	0.96	1.09	0.87	1.05	0.65	0.62	0.57	0.84	1.11	0.7	0.31	0.61	0.92	0.75	1.33	1.45	1.06	0.63	0.85	1.13	1.3	2.02	0.97	2.02	1.48	1.54	1.4	2.02
xylulose	0.65	1.09	0.92	1.21	0.46	0.9	0.68	0.55	0.79	1.19	0.69	1.16	1.11	1.04	1.05	1.79	1.08	0.71	0.35	0.49	0.29	1.2	0.47	1.23	1	0.91	0.86	1.87	0.81	0.83	0.8	1.23								
glucose-6-phosphate (G6P)	1.12	0.96	0.87	0.86	1.18	0.86	1.02	0.9	1.02	1.6	1.19	1.17	2.68	2.48	1.21	1.29	1.14	1	0.49	0.43	1.21	1.01	0.44	0.52	0.54	0.59	0.6	0.24	0.77	0.69	1.63	0.34	0.89	0.97	0.7	0.59	1.19	1.16	1.36	4.45
fructose-6-phosphate	0.97	1.07	0.98	0.88	0.94	0.88	0.86	0.82	0.82	0.96	0.99	0.76	1.07	0.93	1.28	0.91	1.17	0.6	0.57	0.76	1.01	0.59	0.54	0.92	0.69	1.12	0.61	0.51	0.93	0.94	1.17	0.7	1.07	0.81	0.77	0.69	1.06	0.96	1.3	1.58
alpha-ketoglutarate	0.88	0.65	0.29	0.81	0.61	0.55	0.28	1.35	1.59	1.49	0.74	1.48	1.22	1.19	0.33	1.19	0.63	0.74	0.56	0.8	0.83	0.71	0.49	1	0.75	0.41	1.06	0.82	0.66	0.36	0.76	0.76	1.09	1.13	1.02	1.27	1.25	1.25	1.21	1.81
citrate	0.95	1.06	0.9	1.36	0.96	0.96	0.9	1.38	0.82	1.1	1.26	1.27	1.24	1.26	0.54	1.04	2.86	1.69	1.29	1.54	3.07	2.15	1.11	1.77	1.75	2.09	0.89	1	2.2	2.62	0.68	0.75	0.82	0.45	1.13	1.44	1.79	0.75	1.87	3.6
inositol 1-phosphate (I1P)	1.21	1	0.91	0.95	0.94	1.12	0.97	1.03	0.69	1.19	0.76	0.96	1.2	1.18	0.84	0.83	1.02	0.74	0.47	0.34	1.03	0.69	0.39	0.57	0.93	0.68	0.89	0.41	1.06	1.15	1.54	0.68	1.18	0.83	0.89	0.94	0.89	0.94	1.22	1.59
myo-inositol	0.97	1.27	1.72	0.93	1.07	1.18	2.08	1.04	0.99	0.84	1.41	1.53	1.19	0.8	1.09	1.23	0.72	0.83	3.38	1.06	1.66	1.19	3.15	1.36	0.87	1.03	1.1	0.83	1.06	0.81	1.28	0.74	0.96	1.13	0.95	0.96	0.99	1.07	0.77	0.85
adenine	1.01	0.56	0.86	0.76	0.9	0.69	0.63	1.03	0.68	1.32	0.97	1.21	0.92	1.98	1.94	1.11	0.67	0.94	1.08	1.27	0.83	0.81	0.87	1.48	1.04	0.46	1.39	1.4	1.05	0.43	1.73	1.54	1.21	0.7	1	1.19	0.66	0.73	0.82	0.95
adenosine	0.93	0.91	1.26	0.96	1.07	0.93	1.27	1.14	1	0.92	1.08	1.06	1.24	1.02	1.11	1.02	1.15	0.71	0.9	0.8	1.83	0.83	0.91	1.2	0.87	1.09	1.17	1.06	0.97	1.13	1.15	0.94	0.88	0.96	1.09	1.01	1.04	0.95	0.95	1.09
adenosine 5'-monophosphate (AMP)	1.61	1.08	1.17	0.84	1.77	0.78	0.99	0.99	0.79	0.84	0.72	0.75	0.85	0.87	0.99	0.54	0.85	0.34	0.48	0.35	0.82	0.4	0.24	0.47	1.13	0.39	1.03	0.19	1.05	0.5	0.96	0.22	0.74	0.72	0.64	0.87	1.44	0.87	1.86	4.53
guanosine	0.94	0.92	1.04	1.02	0.94	0.99	1.22	1.13	0.81	1.03	1.17	1.06	1.02	1.15	1.08	1.05	0.81	0.84	0.9	0.82	0.73	0.8	0.83	0.84	1.03	0.88	0.91	0.99	1.21	1.1	0.99	0.93	0.75	1.03	0.87	0.93	0.89	1.07	0.99	0.92
allantoin	3.49	2.6	1.07	1.26	2.13	2.36	0.97	1.3	1.12	0.95	1.09	0.75	1.19	1.15	2.34	1.14	3.11	2.11	1.2	1.09	2.09	1.7	1.05	2.13	1.28	2.12	1.65	0.98	1.27	2.64	1.8	1.22	2.27	1.41	4.43	0.99	1.97	1.8	33.24	12.55
uridine	0.89	0.83	1.07	0.81	0.88	0.85	0.94	1.13	1.06	0.88	0.94	1.03	1.24	1.07	1.06	1.13	0.85	0.72	0.59	0.5	1.03	0.95	0.47	0.82	0.87	0.94	1.03	1.32	0.92	0.91	0.96	1	0.8	1.1	1.12	0.88	0.92	1.13	0.94	0.88
cysteine	1.23	0.81	0.77	0.59	0.94	0.98	0.84	0.75	0.46	0.74	0.89	1.47	0.78	0.88	1.04	0.92	0.83	1.2	0.37	0.45	0.55	0.82	0.23	0.57	1.1	0.96	0.85	0.52	0.76	1.27	1.39	0.52	0.8	0.96	0.91	1.06	0.92	0.97	1.3	1.46
glycine	1.71	1.6	1.02	1.13	1.13	1.39	0.98	0.98	0.61	0.49	0.7	0.4	0.76	0.62	1.44	0.5	0.76	0.77	0.17	0.46	0.93	0.59	0.13	0.68	1.02	1.4	1.19	0.65	1.18	2.37	1.88	0.86	0.82	0.72	0.91	0.82	0.7	0.53	1.75	2.58
serine	1.06	1.17	1.03	0.87	1.18	1.39	1.29	1.1	0.62	0.49	0.75	0.6	0.94	0.77	1.58	0.89	0.81	1.01	0.49	0.58	1.17	1.19	0.58	0.77	0.95	0.9	0.85	0.68	1.31	1.48	1.86	0.95	0.81	0.88	0.82	0.51	0.95	1.07	1.93	1.64
S-methylmethionine	1.4	1.41	0.84	0.87	1.38	1.43	0.79	0.89	0.64	0.59	0.71	0.74	0.85	0.86	1.29	0.81	0.86	0.93	0.25	0.37	1.19	0.96	0.38	0.3	1.44	1.6	1.26	0.9	1.45	2	1.89	0.77	0.55	0.72	0.9	1.18	1.01	0.92	2.38	2.93
phenylalanine	1.08	1.48	1.35	1.09	1.19	1.57	1.92	1.19	0.58	0.6	0.7	0.82	0.85	0.79	1.42	1.04	1	1.66	0.63	0.78	1.11	1.69	1.34	0.73	0.97	1.01	1.09	0.77	1.11	1.24	1.81	0.84	0.95	0.81	0.96	0.95	1.36	1.09	2.08	2.74
tyrosine	1.05	1.32	1.13	1.08	1.17	1.46	1.3	1.05	0.69	0.73	0.9	0.67	0.92	0.81	1.55	0.83	0.99	0.91	0.35	0.54	1.1	0.83	0.46	0.56	1.06	0.97	1.01	0.73	1.15	1.27	1.41	0.76	0.89	0.81	0.93	0.89	1.27	1.02	2	2.44
alanine	1.3	1.82	0.9	1.09	1.18	1.91	1.08	1.14	0.34	0.28	0.62	0.26	0.81	0.49	2.26	0.55	0.93	1.27	0.17	0.34	1.4	1.36	0.15	0.54	1.05	1.24	0.77	0.49	1.43	3.05	2.28	0.6	0.89	1.2	0.9	0.92	0.86	1.03	1.16	0.82
asparagine	1.53	1.34	0.99	0.83	1.34	1.42	0.83	0.82	0.62	0.42	0.85	0.5	0.88	0.66	3.22	0.93	0.78	0.84	0.08	0.18	1.27	0.48	0.06	0.33	1.7	1.41	1.16	0.53	1.86	2.42	2.46	0.73	0.46	0.54	0.29	0.77	0.64	0.69	1.08	3.66
aspartate	1.02	1.08	1.02	0.97	1.1	1.07	1.16	0.99	1.01	1.34	1.08	1.79	0.91	1.1	0.51	1.15	0.98	0.98	1.17	1.2	0.86	1.16	1.62	0.91	0.95	0.92	0.97	1	1.09	1.05	1.09	1.02	0.94	0.89	1.01	0.57	0.85	0.81	1.06	0.89
homoserine	1.07	1.73	1.34	0.89	0.8	1.45	1.46	1.16																																
lysine	1.32	1.56	0.66	0.68	1.38	1.6	0.72	0.72	0.52	0.54	0.82	0.94	0.84	0.99	1.54	0.88	0.66	0.96	0.27	0.22	1.05	1.17	0.41	0.22	1.1	1.45	1.18	0.75	1.17	2.16	2.82	0.89	0.88	0.82	0.79	0.82	1.09	0.97	1.6	1.61
methionine	1	1.28	1.12	1.09	0.95	1.19	1.23	1.03	0.64	0.73	0.95	0.72	0.8	0.8	1.1	0.66	0.86	0.81	0.37	0.34	0.85	0.68	0.44	0.48	1.28	0.85	0.9	0.6	1.45	1.04	1.34	0.5	1.24	1.12	0.97	0.93	1.03	1.1	1.06	1.36
arginine	1.57	1.43	0.68	0.44	1.55	1.46	0.72	0.54	0.52	0.4	0.71	0.53	0.96	0.78	2.11	0.62	0.81	0.77	0.11	0.07	1.54	0.99	0.24	0.07	1.3	1.75	1.13	0.53	1.29	2.52	2.75	0.64	0.43	0.31	0.64	1.47	0.85	0.41	1.14	2.16
glutamate	0.9	0.95	0.79	0.87	1.09	1.06	1.21	0.96	1.18	1.34	0.99	1.06	1.15	1.27	0.66	1.6	1.07	0.92	0.74	1.1	1.06	1.04	0.87	0.96	0.83	0.85	0.69	0.92	1.08	0.9	0.89	0.97	0.92	0.91	0.93	0.78	0.99	0.95	1.31	0.98
glutamine	1.08	1.15	1.08	0.99	1.01	1.05	1.06	1.01	0.74	0.86	0.92	0.81	0.82	0.9	1.38	0.92	0.95	0.97	0.4	0.47	0.87	0.87	0.36	0.66	1.44	1.05	1	0.63	1.38	1.23	1.38	0.73	1	1.15	0.97	0.68	0.96	1.06	1.98	2.28
histidine	1.47	1.52	0.61	0.87	1.45	1.57	0.57	0.98	0.58	0.46	0.5	0.63	0.92	0.73	1.5	0.92	0.56	0.78	0.16	0.26	1.11	1.08	0.26	0.26	1.23	1.33	0.73	0.71	1.29	2.04	1.7	0.9	0.44	0.79	0.54	1.03	0.9	0.91	2	4.13
proline	1.05	1.46	1.38	1.17	1.17	1.46	2.14	1.29	0.62	0.62	1.08	1.32	1.59	1.39	1.8	1.94	1.32	1.65	1.38	0.88	1.37	1.68	2.61	1.23	1.14	1.4	1.52	1.44	1.23	1.66	4.79	2.46	0.8	0.77	1.47	1.15	1.15	1.04	3.32	6.19
leucine	1.07	1.41	0.86	1.04	1.13	1.41	0.94	0.96	0.54	0.47	0.62	0.67	0.77	0.68	1.7	0.99	0.81	1.26	0.38	0.49	1.07	1.25	0.33	0.46	1.2	1.17	0.93	0.78	1.31	1.8	1.97	0.8	1.12	0.8	0.84	0.97	1.36	1.01	2	2.63
valine	1.04	1.35	0.99	0.95	1.08	1.36	1.1	0.99	0.59	0.56	0.77	0.7	0.82	0.73	1.45	0.9	1.01	1.27	0.3	0.39	1.17	1.23	0.35	0.59	1.2	1.2	1.12	0.69	1.3	1.7	2.09	0.89	0.87	0.96	0.9	1.05	1	0.91	1.5	1.88
glutathione, oxidized (GSSG)	9.22	1.55	0.92	1.63	18.2	1.42	1.03	1.24	0.31	0.39	1.66	2.17	1.42	0.59	2.08	5.38	1.61	0.72	0.47	0.59	1.21	0.64	0.28	0.8	2.55	1.32	1.18	0.71	2.26	1.56	1.6	0.59	0.87	0.87	0.94	0.81	1.11	0.59	0.7	0.59
glutathione, reduced (GSH)	2.65	3.34	0.9	1.55	2.34	3.3	0.98	1.62	0.69	0.77	0.54	0.65	0.62	0.53	1.13	0.83	0.75	1.37	0.45	0.8	0.96	1.64	0.44	0.85	1.1	0.84	0.49	0.34	0.81	0.89	1.57	0.57	0.98	1.18	1.05	1.59	0.57	1.01	1.21	1.43
1-oleoylglycerophosphoinositol*	0.93	0.87	10.37	1.85	1.01	0.75	8.62	3.38																																
1-palmitoylglycerophosphate	1.12	1.18	2.74	1.07	1.41	1.21	4.08	1.23																									0.54	0.65	1.46	0.66	1.99	0.99	3.3	1.86
1-palmitoylglycerophosphocholine (16:0)	0.66	1.51	2.41	7.48	23.12	1.45	4.01	3.56	1.04	0.7	1.05	1.97	1.7	0.68	0.66	1.23	1.35	0.63	1.2	2.03	1.5	0.73	4.25	5.61	3.77	10.57	1.14	3.59	24.45	12.04	1.84	5.05	1.36	1.01	1	0.73	2.14	1.11	1.17	1.14
1-palmitoylglycerophosphoethanolamine	1.28	1.17	3.57	1.39	1.11	1.07	3.41	2.19	1.1	0.68	1.45	2.25	2.37	0.54	0.63	0.91	1.02	0.94	0.85	0.73	0.98	0.7	1.18	1.46	0.71	1.07	0.92	1.34	0.99	1.44	1.58	1.19	0.94	0.6	1.16	0.46	1.23	1.96	1.98	2.78
1-palmitoylglycerophosphoinositol*	1.19	1.27	5.34	1.13	1.47	1.27	6.37	1.35	0.49	0.92	1.31	3.55	1.33	1.07	0.35	1.07	1.06	1.39	0.65	0.78	0.91	0.89	1.37	2.18	1.02	1.38	1.06	2.11	1.47	2.28	2.65	2.15	0.77	0.39	1.04	0.45	1.62	1.01	2.62	2.27
1-stearoylglycerophosphocholine (18:0)	1	1.37	4.42	1.74	9.35	1.19	5.02	1																									0.9	1.03	0.74	0.62	2.14	1.16	1.59	1.17
2-palmitoylglycerophosphocholine*	1	1.11	2.67	1	2.96	1.37	4.52	1																									2.22	1.4	0.87	0.53	2.44	1.03	0.68	0.47
glycerol 3-phosphate (G3P)	1.37	1.05	1.04	0.68	1.19	1.05	1.18	0.89	0.72	0.95	0.84	0.78	0.91	1	1.11	0.84	0.97	0.83	0.71	0.64	0.81	0.86	0.75	0.92	0.78	0.88	1.01	0.56	1.08	1.28	1.56	0.72	1.17	1	0.97	0.61	0.91	0.83	0.96	0.99
phosphoethanolamine	1.08	0.89	0.5	0.63	1.18	0.84	0.61	0.6	0.87	1.06	0.78	0.72	0.83	1.08	0.94	0.85	0.76	0.67	0.51	0.49	1.17	0.87	0.61	0.39	1.02	0.98	1.2	0.5	1.26	1.3	1.16	0.58	0.88	0.56	1.02	0.21	0.98	0.76	2	1.4
phytosphingosine	0.54	1.26	2.22	3.64	19.27	1	2.4	2.17	0.91	1.09	0.86	1.1	1.18	1.2	0.8	0.83	0.97	0.47	2.29	1.44	1.1	0.7	5.53	1.44	2.49	7.67	0.94	2.62	13.07	8.12	0.92	1.88	0.55	0.56	1.1	0.53	2.02	1.33	2.77	1.88
sphinganine	0.39	1.22	3.22	9.53	26.07	0.9	3.48	5.8	0.92	1.04	1.03	0.97	1.97	1.23	1.22	0.88	1.17	0.81	7.92	2.09	1.29	0.72	33.74	4.05	2.58	8.48	0.98	1.8	13.99	7.49	0.87	1.12	0.92	0.94	1.71	0.67	2.5	1.68	4.32	2.06
beta-sitosterol	1.13	1.17	1.13	1.1	1.25	1.19	1.22	1.1	0.95	1	1.02	1.23	1.2	0.96	0.97	1.13	0.91	1.14	1.11	1.1	1.04	1.29	1.12	0.94	1.04	0.84	1.03	1.22	1.33	1.19	1.1	1.04	0.92	0.9	0.91	1.08	1.04	0.99	1.02	1.14
